# Vegetation trends over eleven years on mountain summits in NW Argentina

**DOI:** 10.1002/ece3.4602

**Published:** 2018-11-14

**Authors:** Julieta Carilla, Stephan Halloy, Soledad Cuello, Alfredo Grau, Agustina Malizia, Francisco Cuesta

**Affiliations:** ^1^ Instituto de Ecología Regional Universidad Nacional de Tucumán—Consejo Nacional de Investigaciones Científicas y Técnicas (CONICET) Tucumán Argentina; ^2^ Ministry for Primary Industries Wellington New Zealand; ^3^ Instituto de Química del Noroeste (INQUINOA) Tucumán Argentina; ^4^ Biodiversity Department Consorcio para el Desarrollo Sostenible de la Ecorregión Andina (CONDESAN) Quito Ecuador; ^5^ Palaeoecology and Landscape Ecology, Institute for Biodiversity and Ecosystem Dynamics (IBED) University of Amsterdam Amsterdam The Netherlands

**Keywords:** Andes, climate change, community turnover, elevation gradient, GLORIA initiative, plant diversity

## Abstract

As global climate change leads to warmer and dryer conditions in the central Andes, alpine plant communities are forced to upward displacements following their climatic niche. Species range shifts are predicted to have major impacts on alpine communities by reshuffling species composition and abundances. Using a standardized protocol, we surveyed alpine plant communities in permanent plots on four high Andean summits in NW Argentina, which range from 4,040 to 4,740 m a.s.l. After a baseline survey in 2006–2008, we resurvey the same plots in 2012, and again in 2017. We found a significant decrease in plant cover, species richness, and diversity across the elevation gradient in the three censuses and a strong decrease in soil temperature along the elevation gradient. We found a high plant community turnover (37%–49%) among censuses, differentiating according to summits and aspects; major changes of community turnover were observed in the lowest summit (49%) and on the northern (47%) and western (46%) aspects. Temporal patterns in community changes were represented by increases in plant cover in the highest summit, in species richness in the lower summit, and in diversity (Shannon index) in the four summits, over time, together with increase in small herbs and non‐tussock grasses. We suggest that the observed trend in plant community dynamics responds to short‐term temperature and precipitation variability, which is influenced by El Niño Southern Oscillation (ENSO), and due to time lags in plant community response, it may take much longer than one decade for the observed trends to become stables and statistically significant. Our study provides an important foundation for documenting more profound changes in these subtropical alpine plant communities as global climate change continues.

## 1 INTRODUCTION

High mountain ecosystems are sensitive to climate change, mainly because their organisms are governed by low‐temperature conditions (Halloy, 1989; Körner, 2012; Pauli et al., 2012). High mountain ecosystems are often less affected by direct anthropogenic land use impacts and biotic factors, such as competition, which decrease with altitude (Llambi, Law, & Hodge, 2004; Mark et al., 2015), as environmental stress increases (Anthelme, Meneses, Valero, Pozo, & Dangles, 2017; Halloy, 1989). Thus, high mountain ecosystems can be considered “natural experiments” to study the impact of climate change on vegetation, and useful for global scale comparisons (Dangles et al., 2017). GLORIA (Global Observation Research Initiative in Alpine Environments) is an international network of long‐term monitoring sites that have been developed to assess the response of vegetation to climate change and compare it across high mountains all over the world (Gottfried et al., 2012; Pauli et al., 2012). Within GLORIA, the GLORIA‐Andes Network links sites in Andean countries (Cuesta et al., 2012, 2017 ).

Climate change is one of the main factors modifying high mountain vegetation and may result in species migration, adaptation, or extinction in the coming decades (Hoegh‐Guldberg et al., 2008; Parmesan, 2006; Pauli, Gottfried, Reiter, Klettner, & Grabherr, 2007). A reconstruction based on tree rings from the high Andes of NW Argentina showed a consistent aridity trend for the last decades with an increase in the frequency of drought events (Morales, Carilla, Grau, & Villalba, 2015). The observed trend of increasing aridity along with projected temperature increases (Urrutia & Vuille, 2009; Vuille et al., 2017) will make high mountain summits of NW Argentina too dry and warm for alpine plant specialist (cryophilic species), and therefore, they may undergo a range contraction and/or become locally extinct. Evidence for upward displacement of species and increase in community richness at high altitudes has been recorded in different mountain ranges of Europe (Dullinger et al., 2012; Erschbamer, Kiebacher, Mallam, & Unterluggauer, 2009; Grytnes et al., 2014; Pauli et al., 2012; Wipf, Stöckli, Herz, & Rixen, 2013), Africa (Hemp, 2009), and South America (Moret, Aráuz, Gobbi, & Barragán, 2016; Morueta‐Holme et al., 2015; Seimon et al., 2007, 2017 ), and also differences in species growth among summits aspects (Sklenář, Kučerová, Macková, & Romoleroux, 2016). Further, population decline and losses in genetic diversity due to warming driven range reduction have been observed in narrow‐range tropical alpine species (Chala et al., 2016). In addition, shifts in species dominance and plant cover may occur, which is the most detectable change in short‐ to medium‐term studies (Halloy, 2002; Seimon et al., 2009; Yager, Resnikowski, & Halloy, 2008).

Long‐term trends are often masked by short‐term dynamics and variability (Perez et al., 2010). Historic records and multidecadal time series are needed to understand the natural range of variability (Landres, Morgan, & Swanson, 1999) and assess recent trends in ecosystems and biodiversity changes. Continued observations of vegetation dynamics (changes in species composition and cover) in the high Andes encompass short periods of monitoring, most of them from the beginning of the 21st century (Cuesta et al., 2012, 2017 ). Few historic records and environmental proxies provide a wider perspective of the natural variability of the ecosystem versus changes caused by human activities (Halloy, 2002; Morales et al., 2015; Morueta‐Holme et al., 2015).

Ground truthing through repeated surveys in permanent plots is essential to answer the following questions: How is the distribution of alpine vegetation affected by climate change across an elevation gradient? Will climate change cause species to shift their range upward in elevation? Is it possible to detect changes in alpine plant community's composition after 10 years of continuous observation? The aim of this study was to analyze alpine plant species composition along summits and aspects, and to assess plant community dynamics over a period of 9–11 years (from 2006/08 to 2017) on four summits, ranging from 4,040 to 4,740 m a.s.l., in Cumbres Calchaquíes, Tucumán, Argentina, in order to establish the foundations for understanding the long‐term trends from vegetation patterns of short‐term variability. To explain the observed vegetation patterns and dynamics, we related vegetation variables and plant life forms with soil temperature. We hypothesized that (1) temperature regime, controlled by elevation and aspect is the main factor determining the prevailing vegetation and the distribution of plant species, and (2) even short‐term thermal changes (over several years) lead to a shift in species composition, and changes in plant cover and diversity.

## 2 METHODS

### 2.1 Study area

**Figure 1:**
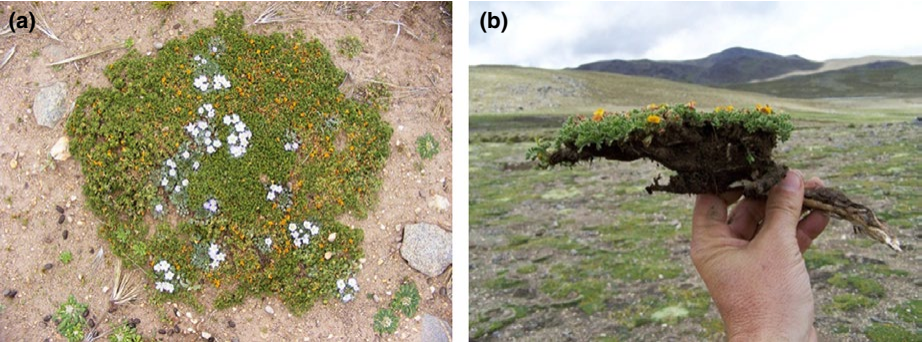
Plant community known as cryptofruticetum, (a) composed by flattened vegetation, with great diversity of tiny colorful flowers in summer. (b) Adesmia crassicaulis (in the hand) one of the species of the cryptofruticetum community observed in the ground, in Cumbres Calchaquíes, Tucumán, Argentina

A GLORIA target region was established in Parque Provincial Cumbres Calchaquíes, Tucumán, NW Argentina (26°40’ S 65°44’ W; Lomáscolo et al., 2014) in 2006–08. Geologically, this mountain range belongs to the Pampean system and is characterized by a plateau at 4,300 m a.s.l., with dozens of lakes of glacial origin, referred to as Huaca Huasi lakes. The dominant bedrock is Precambrian metamorphic rocks, from solid crag to quaternary moraine accumulation (Halloy, 1985a). The area is within the High Andean ecoregion (Cabrera, 1976), Calchaquí subdistrict with high levels of plant diversity and restricted distribution ranges (Halloy, 1985b). There are three main vegetation communities dominated by (a) Festuca ortophylla (Poaceae, Iro grasslands); (b) ground‐hugging plants such as Adesmia crassicaulis (Fabaceae), Tetraglochin inerme (Rosaceae), and Pycnophyllum convexum (Caryophyllaceae), a community known as cryptofruticetum (up to 20 species per 1m^2^; Figure 1); and (c) typical wetland vegetation communities of cushion bogs (Halloy & Laurent, 1988; Halloy, 1985a). This area presents some advantages for long‐term studies, such as vegetation records since 1976 (Halloy, 2002), lake level records going back to the 1980s (Casagranda, 2010; Halloy, 2002), discontinuous soil temperatures since the 1970´s (Halloy & Laurent, 1988; Halloy, 1983, 1985a) and a detailed physical characterization of the area. The main land uses are recreation (hiking) and cattle grazing at the lowest summit. Herbivory by native camelids (Lama guanicoe) and big rodents (Lagidium viscacia) is present in the whole area, although the limited accessibility and isolation has favored ecosystem conservation.

Direct measurement of annual precipitation at 4,250 m a.s.l. are available for 1977–1978, recording c. 385 mm on average, mainly as snow, concentrated in the summer months (December to March), and mean annual air temperature was estimated as 1.5ºC (Halloy, 1985a). A recently installed meteorological station at 4,200 m a.s.l., recorded an annual precipitation of 333 mm and an average temperature of 2.9°C in 2016.

### 2.2 Sampling design, data recording, and preparation

**Figure 2:**
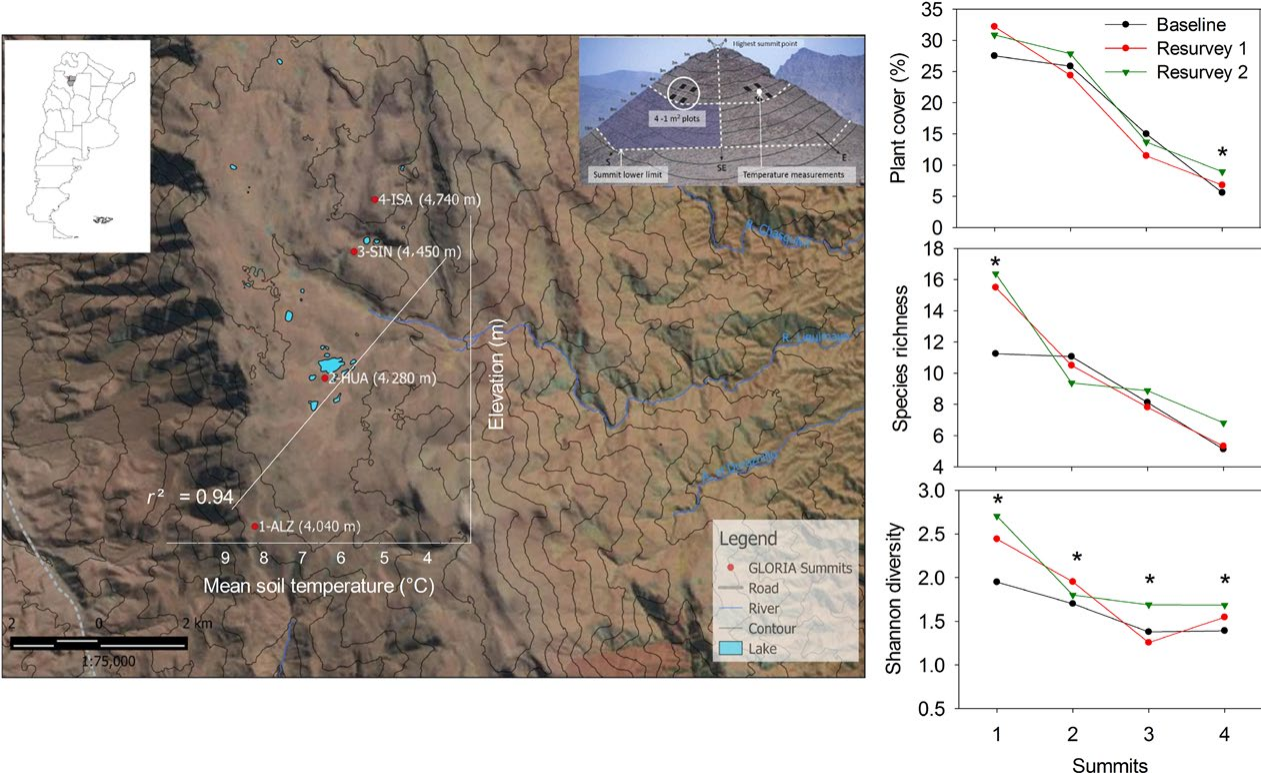
Study area. GLORIA summits indicated with a red point in a Landsat image. Tucuman, Argentina map (left corner). Sampling design scheme showing 1 m^2^ plot, the position of data loggers and summit lower limit (right corner, from Pauli et al., 2015). Linear regression between mean soil temperature and elevation is superposed with the image (represent the main hypothesis). The main spatial and temporal trends in vegetation variables are represented by graphs in the right; symbols indicate increasing trends over time

Summit site setup and vegetation surveys followed GLORIA sampling methodology (Halloy, Ibáñez, & Yager, 2011; Pauli et al, 2015). Four summits were selected at different altitudes, with the same bedrock and under the same regional climatic conditions. Summits were coded from lower to higher altitude as: 1‐ALZ, 2‐HUA, 3‐SIN, and 4‐ISA (Figure 2). Baseline sampling was carried out in February 2006 (2‐HUA), March 2007 (1‐ALZ and 3‐SIN), and January 2008 (4‐ISA), the first re‐survey, was carried out in February (1‐ALZ, 2‐HUA, and 3‐SIN) and December 2012 (4‐ISA) and the second re‐survey in February 2017.

We recorded vascular plant species and their relative percentage cover, lichen, and bryophyte cover (total), and substrate cover: solid rock (fixed in the ground), gravel/scree (debris material), bare soil (including clay and sand), organic matter, and feces (from camelids and rodents in all summits, and cattle in the lower summit). Vascular plants were classified into life forms, following Ramsay and Oxley (1997) adapted for the GLORIA—Andes network, as shrubs (including subshrubs), cushion plants (lax and hard cushions), tussock grasses, non‐tussock grasses (cespitose and tufted grasses), erect herbs, prostrate herbs (including trailing and creeping herbs), rosettes (including basal and acaulescent), and ferns.

We estimated species composition and richness at 1) summit scale based on semiquantitative criteria for visual estimation, by subdividing the summits in sections and walking them thoroughly, at the baseline survey, and by using flexible points and areas method (PAF; Halloy et al., 2011), for the resurveys. PAF method is useful for quantifying species cover in relation to a total area with minimum sampling effort. However, in this study, we used PAF in order to record the complete species list of each summit. The lower limit of the summit was established at 10 m vertically down from the highest summit point (HSP). We measured species richness and plant cover at 2) 1 m × 1 m plots, with four plots for each aspect (north, east, south, and west, resulting in 16 plots for each summit). Each 1 m^2^ plot was subdivided into 100 subplots of 0.01 m^2^, equivalent to 1% cover with 100 crosshair points.

We estimated species diversity using the Shannon‐Weaver index (H`), and species turnover rate as a measure of community change, with the formula: (Species gain + species lost)/(initial richness + species gain) × 100 (in 11 years), following Ramirez‐Villegas et al. (2014). Plant cover, Shannon diversity index, and species turnover were only calculated at plot scale, where species abundance data was more accurate (Zimmer, Meneses, Rabatel, Soruco, & Anthelme, 2014). New and missing species were reported only for the summit area.

Botanical nomenclature was verified in herbarium of National University of Tucuman (LIL collection), in several taxonomical meetings and in uploaded Flora Argentina (Darwinion Botanical Institute; www.darwin.edu.ar/Proyectos/FloraArgentina/) and in TROPICOS (www.tropicos.org).

### 2.3 Soil temperature records

We measured soil temperature with data loggers (tidbit, Onset) at four points (north, east, south, and west) on each summit, located at 5 m vertically down from the HSP at 10 cm below the soil surface, with a recording frequency of every two hours (i.e., 12 records per day). Two data loggers failed at the beginning of the project (1‐ALZ north and 3‐SIN east), thus we used records from 14 data loggers, for the period March 2009 to February 2012 (36 months). From the dataset for 2009–2012, we derived the following seven temperature variables: mean soil temperature, minimum average soil temperature, and maximum average soil temperature, calculated as the mean/minimum/maximum monthly average over the days, mean soil temperature for the growing season (October to March) and for non‐growing season (April to September). We also calculated daily and seasonal thermal amplitude (maximum ‐ minimum daily/monthly soil temperature). We used a correlation matrix to avoid collinearity; mean and minimum average soil temperature were highly correlated with elevation (*r* ^2^ = 0.9; Supporting Information Table S1; Figure 2). From the 14 data loggers, seven recorded until February 2015, allowing us to compare these seven series with vegetation variables from the same sampling units (1‐ALZ west, 1‐ALZ south, 3‐SIN west, 3‐SIN south, 4‐ISA east, 4‐ISA south, 4‐ISA west), using mean and minimum average soil temperature. To associate temperature records with field surveys, we used soil temperature data from 2009–2010 as a baseline, 2012–2013 for resurvey 1, and soil temperature data from 2014–2015 for resurvey 2. Finally, we used the annual mean monthly Southern Oscillation Index (SOI) as a proxy of precipitation (data available from https://www.bom.gov.au/climate/current/soihtm1.shtml).

### 2.4 Data analysis

In order to assess temporal trends in temperature series data, we used linear regression, analyzing each data logger record for 2009–2015 period (seven data loggers; function lm in R; Chambers, 1992). We explored the relationship between the seven temperature variables with elevation using linear regression (function lm in R) and aspects using two‐way ANOVA (function aov in R. Chambers, Freeny, & Heiberger, 1992).

We analyzed trends over three vegetation metrics (variables): (a) vascular plant cover (total cover of all vascular plant species; plant cover hereafter), (b) vascular species richness (species richness hereafter), and (c) Shannon‐Weaver diversity index (Shannon index hereafter), across the elevational gradient and examined the relationship between temperature and vegetation metrics from the 1 m^2^ plots (*N* = 56; 14 plots of 1 m^2^ per summit, and per censuses), with linear regression using the function lm in R (Chambers, 1992; excluding failed data loggers 1‐ALZ N and 3‐SIN E).

In order to assess the relation between temporal trends in vegetation variables and soil temperature, we related soil temperature records for 2009–2015 period (seven data loggers) with the three vegetation variables for the same seven sampling units (function lm in R; Chambers, 1992). For both, soil temperature and vegetation variables we explored absolute records and data anomalies, calculated as soil temperature/vegetation metrics in a census year minus soil temperature/vegetation metrics average of the three censuses divided by standard deviation.

We used linear mixed models and generalized linear mixed models (LMM and GLMM; Bolker et al., 2009) to analyze variability in plant cover, species richness, and diversity index among aspects within summits, among summits, and among censuses, with aspect and census as fixed effects and summit as a random effect. For model construction, we considered Gaussian distribution for plant cover (square root transformed data) and Shannon index, using the package lme4 in R (Bates, Kliegl, Vasishth, & Baayen, 2015), and followed by lmerTest (Kuznetsova, Brockhoff, & Christensen, 2017), to obtain p values for fixed effects, and Poisson distribution for species richness using the package GLMM (Knudson, 2016).

To assess compositional similarity among summits, we used non‐metric multidimensional scaling (NMDS) to visualize differences between each combination of summit, aspect, and year. We created a matrix based on the total percent cover of each species in the four 1 m^2^ plots within each aspect/summit combination in each year (95 species in four aspects × four summits × three surveys = 48 points). We calculated the Bray–Curtis distance (Legendre & Legendre, 1998) between these summit—aspect—year totals after taking the square root of the raw percent cover data, which gives more weighting to low‐abundance species (McCune & Grace, 2002). For the NMDS and the Bray–Curtis distance, we used PC‐ORD 5.0 (McCune & Mefford, 1999). We indicated trajectories in species composition over the three surveys with “successional vectors” represented by arrows. For Poaceae species, we used the genus level taxa to avoid the noise from uncertainties in taxonomical identification. Therefore, the 31 species of Poaceae were lumped into eight genera for the ordination analysis (Supporting Information Appendix S1). We used a two‐dimensional configuration with a final stress of 16 which indicates a quite satisfactory agreement between graph configuration and the similarity matrix (Legendre & Legendre, 1998; McCune & Grace, 2002), and it was significantly different from chance (Montecarlo: 250 runs with randomized matrix, *p* = 0.002). We report the percentage of variance represented by each axis on the *r* ^2^ of the relationship between distance and original space (McCune & Grace, 2002). In order to relate plant community composition with biotic and abiotic factors, we related the ordination diagram with a secondary matrix of 22 variables: the seven variables related to soil temperature (mean, min, max, mean growing season, mean non‐growing season, daily, and seasonal amplitudes), percent cover of the five substrate cover types, eight life forms categories and bryophytes, and lichen cover. We computed Kendall tau correlations between plot scores and species cover (main matrix) to identify species that were driving the ordination, and between plot scores and secondary matrix (McCune & Mefford, 1999). We computed Bonferroni correction as 0.05/95 (main matrix) and 0.05/22 (secondary matrix), which gives *p* < 0.0005 and 0.002, respectively, as significant (Legendre & Legendre, 1998).

We calculated the relative changes in vegetation variables in 9/11 years as the difference between the second resurvey and the baseline, divided by the baseline value. We used Kruskal Wallis nonparametric tests (Sokal & Rohlf, 1995) to test for differences in the relative changes of vegetation variables among summits and Friedman non – parametric test for dependent samples (Siegel, 1956) to test for differences in percentage cover of life forms categories in the 9/11 years between summits, and between census years across summits.

## 3 RESULTS

### 3.1 At the summit scale

#### 3.1.1 Changes in species number and composition

Across the entire area of the four summits (1‐ALZ, 2‐HUA, 3‐SIN, 4‐ISA; 21.855 m^2^), and the three censuses (2006/08, 2012 and 2017) we recorded 139 species, belonging to 80 genera and 35 botanical families (Supporting Information Appendix S1). The most represented families were Asteraceae with 22% and Poaceae with 20% of the total species pool. Poaceae was also the dominant family in terms of plant cover. We recorded 125 species in the baseline survey, 131 species in resurvey 1 and 114 in resurvey 2. In the 9/11 years of analysis, 14 species were newly recorded, and 13 species were no longer recorded. The number of new species arriving from the baseline to resurvey 2 decreased with altitude (Table 1), with 14 new species in the lowest summit 1‐ALZ, most of them on the eastern and southern aspects. Half of the 14 new species in 1‐ALZ had already been recorded at baseline in at least one other summit, while the remaining seven species were not previously recorded on any of the four summits. The number of missing species was also higher on the lower summit (17), and on the northern aspect (27). On the highest summit, 4‐ISA, the number of missing species (15) was higher than the number of new species (5). Of the 35 botanical families recorded at the baseline, three (Alstroemeriaceae, Crassulaceae, and Rubiaceae) were not recorded in resurvey 2, while one family (Cyperaceae) appeared in resurvey 2 for the first time (Table 1; Supporting Information Appendix S1, Data S1).

**Table 1:** Characteristics of the Cumbres Calchaquíes GLORIA pilot site: data for vascular plants in baseline (BL), first (RS1) and second (RS2) resurvey. Species number, species turnover (species gain + species lost/initial richness +species gain) × 100), number of botanical families, plant cover (%), and Shannon diversity index (H). For species number and family numbers values refer to the entire summit area and the summit area sectors or each aspect. For species richness, turnover, plant cover, and Shannon index, values are referring to the 1 m^2^ plot. For new and lost species, Poaceae were not considered

Summit code	Number of species at summit scale						Number of species at plot scale (mean number of species in 1 m^2^)				Species Turnover	*N* botanical families			Plant cover (%)			Shannon diversity index		
	BL	RS1	RS2	Total	New	Lost	BL	RS1	RS2	Total		BL	RS1	RS2	BL	RS1	RS2	BL	RS1	RS2
1‐ALZ	77	89	71	99	14	17	32 (11)	55 (16)	49 (16)	62	48.8	32	31	29	27.5	32.2	30.9	1.95	2.44	2.68
2‐HUA	68	68	64	83	8	12	45 (11)	44 (11)	36 (9)	57	37.1	23	21	22	25.8	24.4	27.9	1.76	1.94	1.78
3‐SIN	69	69	59	83	5	14	37 (8)	42 (8)	42 (9)	59	42.7	25	24	21	15	11.5	13.7	1.38	1.22	1.71
4‐ISA	55	44	41	61	5	15	29 (5)	26 (5)	24 (7)	36	37.6	20	18	18	5.6	6.8	8.9	1.39	1.55	1.68
*N*	115	109	92	132	10	27	52 (11)	58 (10)	55 (11)	74	46.7	33	31	27	16.2	17.5	23.2	1.93	2.16	2.30
E	97	101	92	118	13	20	47 (11)	60 (12)	58 (12)	75	34.5	31	32	30	25	19.5	24.6	1.68	2.02	2.06
S	93	93	80	122	13	22	46 (7)	56 (7)	52 (8)	70	38.5	28	29	26	16.5	17.6	19.4	1.31	1.14	1.36
W	102	102	82	119	9	22	51 (9)	64 (11)	60 (11)	74	46.5	31	30	28	16.1	20.4	17.7	1.56	1.83	2.14
Total	125	131	114	139	14	13	88 (9)	96 (10)	87 (10)	109	41.5	34	34	33	18.45	18.75	21.2	1.62	1.79	1.96

### 3.2 At the 1 m^2^ plot scale

#### 3.2.1 Species number, composition, and cover

We recorded a total of 109 vascular plant species belonging to 73 genera and 30 botanical families in the 64 plots on the four summits over the three censuses (Supporting Information Appendix S1), with an average of 10 species per plot, ranging between 5 to 16 species per plot (Table 1). Taking into account the three censuses, the most abundant species overall in terms of their cover were *Festuca orthophylla* (5.4%) and *Pycnophyllum convexum* (4.5%), with varying abundance between summits. The dominant species in 1‐ALZ were *Deyeuxia colorata*,* Jarava leptostachia*, and *P. convexum* (7.4, 4.9, 4.3%, respectively), in 2‐HUA was *F. ortophylla* (18%), in 3‐SIN, were *P. convexum* and *Festuca uninodis* (6.3% and 5%, respectively), and in 4‐ISA were *P. convexum* and *Mulinum axiliflorum* (3.1% and 2.6%, respectively). The most abundant species in each aspect (averaged across the three censuses) were: in the north, *Azorella compacta* and *P. convexum* (with 4.8% and 3.8%, respectively); in the east, *P. convexum*,* A. compacta*, and *Parastrephia lucida* (with 7.1%, 5.8% and 5.1%, respectively); in the south, *P. convexum* and *F. orthophylla* (9.7% and 4.7%, respectively) and in the west were *F. orthophylla, D. colorata, and Tetraglochin inerme* (4%, 3.5% and 3.1%, respectively).

#### 3.2.2 Soil temperature and Southern oscillation index

Soil temperature averaged across the 14 data loggers did not show a significant trend over the three years (*r* ^2^ < 0.1; March 2009 to February 2012). From the longer time series (2009–2015) available from seven data loggers, five showed a positive trend in minimum soil temperature (1‐ALZ west, 3‐SIN south, 4‐ISA east, 4‐ISA south, 4‐ISA west, with *R* ^2^ = 0.6, 0.2, 0.3, 0.2, 0.5, respectively), whereby 1‐ALZ west was marginally significant (*p* = 0.06), and four showed a positive trend in mean temperature (1‐ALZ south, 3‐SIN west, 3‐SIN south, 4‐ISA west, with *R* ^2^ > 0.2). The highest soil temperatures were recorded in 2012–2013 and 2014–2015, while the lowest was in 2011–2012. Differences between the last (2014–2015) and the first (2009–2010) minimum temperature recorded of the six‐year series were positive for the three summits, with an increment of 0.52°C. Spatially, soil temperature decreased with elevation, as expected; mean annual soil temperature on the lowest summit was 3.5°C warmer than the highest summit (1‐ALZ, 7.9°C, 4‐ISA, 4.4°C), similar difference for the minimum average soil temperature (1‐ALZ, 0.9°C; 4‐ISA, −2.8°C). Seasonal variability showed a bimodal curve, with two warm peaks, in November and January and the coldest month in July. Mean soil temperature for growing season was highest in 1‐ALZ at 12°C, decreasing linearly with elevation (*R* ^2^ = 0.6, *p* = 0.002; Table 2). Temperature also differed among aspects; east and north facing slopes showed the highest mean soil temperature (c. 2°C warmer than west and south aspects; *F*(3, 274,586) = 856.5, *p* < 0.001), and the highest maximum average soil temperature (c. 3–5°C warmer than western and southern aspects; Table 2), as well as the highest daily thermal amplitude (*F*(3,164) = 9.92, *p* < 0.001). The annual mean monthly Southern Oscillation Index (SOI) also showed high variability over time, with the highest values in 2010–2012 and the lowest in 2015–2016. In general wetter years were colder and dryer years were warmer, indicated by the negative relationship between SOI and minimum and mean soil temperature (*R* ^2^ = 0.6 and 0.7, respectively; Supporting Information Figure S1).

**Table 2:** Temperature data (°C) obtained from data loggers at 10 cm below the soil surface for the 2009–2012 period. Summit temperature is the monthly average temperature at the four aspects and aspect temperature is the monthly average across the four summits (mean + standard deviation). Daily thermal amplitude is the mean difference between maximum and minimum temperature each day. Seasonal thermal amplitude is the mean difference between growing season and non‐growing season temperature. Note that two data loggers (1ALZ north and 3‐SIN east) failed, and therefore no data are presented

Sites	Temperature (annual)			Mean temperature		Daily thermal amplitude	Seasonal thermal amplitude
	Min	Mean	Max	Growing season	Non‐growing season		
1‐ALZ	0.9 + 3.9	7.9 + 4.9	16.9 + 6.9	12.2 + 1.5	4.1 + 3.2	7.0 + 3.4	8.1 + 1.9
2‐HUA	−0.2 + 3.6	7.3 + 3.8	17.3 + 5.6	10.5 + 1.1	4.1 + 2.7	10.1 + 3.7	6.4 + 1.8
3‐SIN	−2.2 + 4.1	5.4 + 3.9	15.4 + 5.5	9.3 + 1.3	0.8 + 3.1	8.4 + 3.4	8.5 + 1.9
4‐ISA	−2.8 + 4.3	4.3 + 4.7	14.7 + 7.5	8.3 + 1.1	1.8 + 2.6	8.3 + 3.2	6.4 + 1.7
N	−2.0 + 4.1	6.9 + 3.2	18.8 + 4.5	9.9 + 1.1	2.3 + 3.0	10.4 + 2.3	7.6 + 2.2
E	−0.4 + 4.3	7.5 + 4.9	17.9 + 7.0	11.0 + 1.1	5.4 + 2.9	10.3 + 2.3	5.6 + 1.8
S	−0.8 + 4.0	5.2 + 5.1	15.4 + 6.0	9.2 + 1.5	1.0 + 2.7	7.0 + 2.5	8.2 + 1.3
W	−1.3 + 3.6	5.7 + 4.0	13.2 + 7.7	10.0 + 1.2	2.8 + 2.8	7.6 + 1.8	7.2 + 1.1

#### 3.2.3 Spatial pattern of vegetation, substrates, and temperature

**Figure 3:**
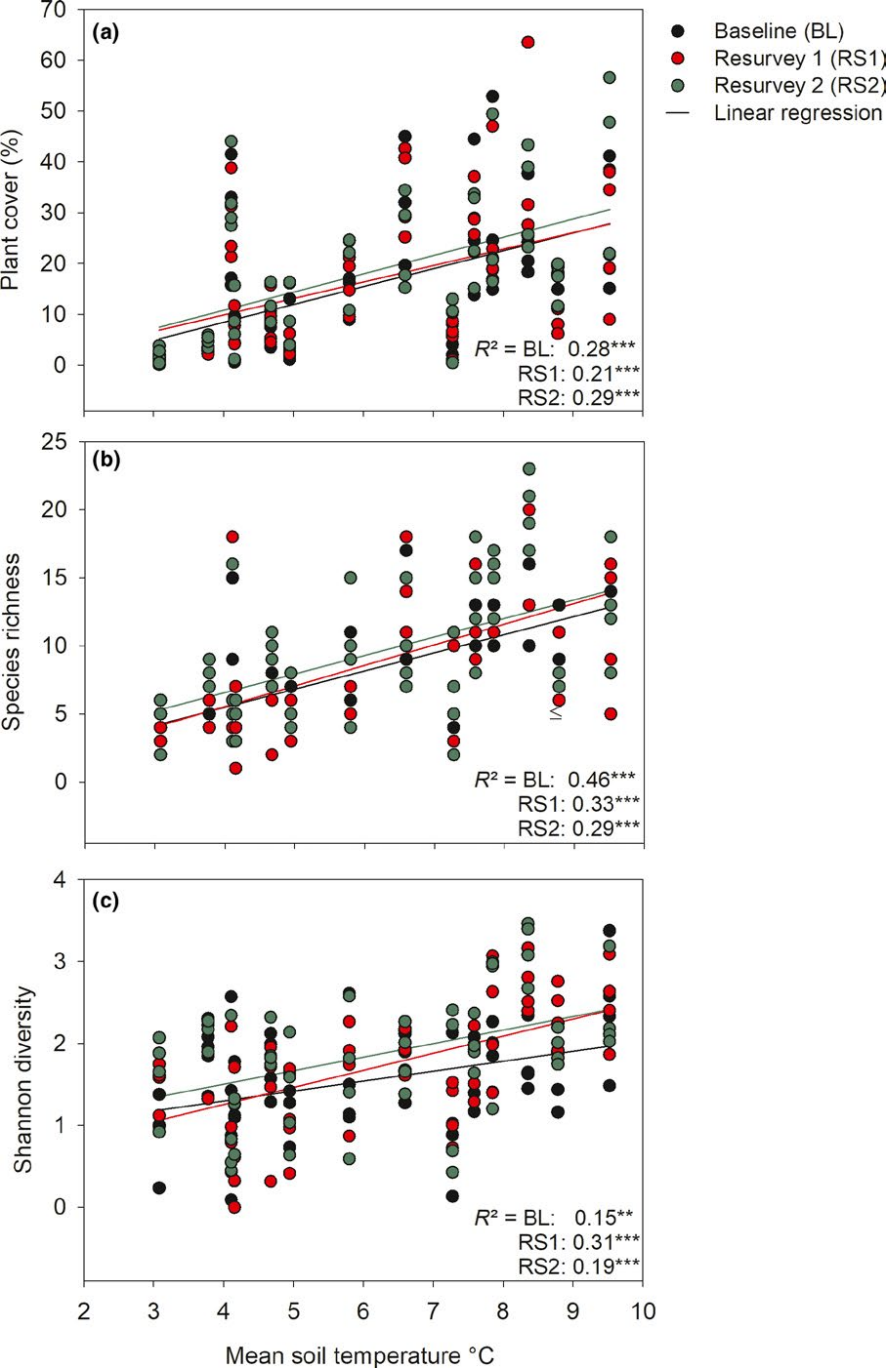
Scatterplot of (a) plant cover, (b) species richness, and (c) Shannon diversity index at plot scale in relation to mean monthly soil temperature for baseline, first, and second resurveys. Each point represents and individual 1 m^2^ plot (four units per aspect except 1‐ALZ north and 3‐SIN east‐ 56 per census). *** *p* < 0.001, **p < 0.01

Plant cover, species richness, and Shannon diversity significantly increased with mean soil temperature in the three censuses (Figure 3a–c), as well as, with minimum average soil temperature. The three vegetation metrics showed significant differences among aspects within summits, among summits, and surveys: plant cover, species richness, and diversity were highest in the lower summits, with 28% of plant cover, 12 species per m^2^ on average (with a maximum of 23 species in 1‐ALZ) and diversity index of 2.4, averaged across the three censuses. The highest plant cover tended to occur on eastern aspects, whereas lower species richness and diversity index tended to occur on the southern aspect, the colder side of the summits (Supporting Information Table S2). Census (time of survey) had a significant positive effect on diversity (in the four summits) and richness (in the lower summit; Supporting Information Table S3 for LMM and GLMM models).

Relating vegetation with soil temperature across the three censuses (the seven plots with the longer temperature series from 2009 to 2015), we found a significantly positive correlation between plant cover, species richness, and Shannon diversity with mean and minimum average soil temperature (*N* = 21; seven data loggers and three censuses; *R* ^2^ from 0.4 to 0.95, *p* < 0.05. Supporting Information Figure S2).

Organic matter cover was significantly higher in the lower summits (1‐ALZ and 2‐HUA; KW (3, 192) = 74.86, *p* < 0.001), and in the southern aspect (KW (3, 192) = 18.53, *p* < 0.001), where tussock grasses dominated, and presented a positive relationship with vegetation metrics (in average; *R* ^2^ = 0.2–0.5, *p* < 0.05). Rock cover, showed the opposite pattern than organic matter, being higher in the highest summits (KW(3,192) = 93.58, *p* < 0.001), in the northern aspect KW(3,192) = 15.14, *p* = 0.002), and presented a stronger negative relationship with vegetation metrics (*p* < 0.05) for species richness (*R* ^2^ = 0.72) and vegetation cover (*R* ^2^ = 0.54). Gravel cover was higher in the western aspects (KW(3,192) = 59.74, *p* < 0.001, mainly in higher summits), with no clear relationships with vegetation metrics. Organic matter, rock, and gravel cover did not change over time.

#### 3.2.4 Patterns in species richness, plant cover, and composition over 11 years

**Figure 4:**
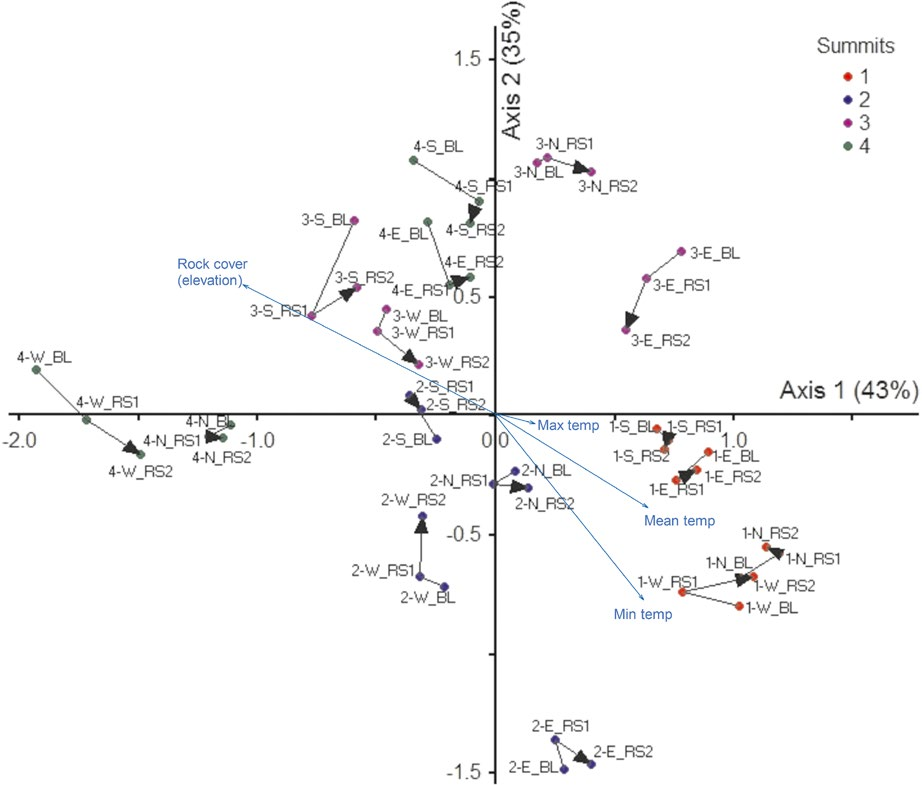
Non‐metric multidimensional scaling (NMDS) based on a 48‐sampling units x 95‐vascular species cover matrix. Each point represents an individual summit/aspect/year combination, where the species data from the 4 1 m^2^ plots in each summit‐aspect were pooled. Axis scale is proportional to variance contribution explained. Black arrows represent successional trajectories over time (Baseline: BL ‐> Resurvey1: RS1 ‐> Resurvey 2: RS2) by aspect: north (N), east (E), south (S), west (W). Main environmental and topographic factors are indicated with blue arrows (obtained from correlation with secondary matrix)

Plots tended to segregate along two NMDS dimensions based on vascular plant composition for different summits and aspects in three censuses (Figure 4). The two axes represented 78% of the variance in species composition (axis 1, 43% and axis 2, 35%). Summits were clearly separated by soil temperature, mainly mean and minimum (and thus elevation); lower summits (associated with positive axis 1 and negative axis 2) presented higher plant cover and richness in species and life forms, and were more homogenous within aspects (lower coefficient of variation comparing scores between aspects in both axis), than the highest summits, associated with positive axis 2 (3‐SIN) and negative axis 1 (4‐ISA), where rock cover predominates (Figure 4). Of the 95 species, 18 species were positively correlated with axis 1, and four with axis 2, four species were negatively correlated with axis 1, and 11 species with axis 2 (Supporting Information Table S4). The main secondary variable driven the ordination were soil temperatures (mean, min and max), which were associated with total plant cover (positive correlation with axis 1 and negative with axis 2) and thus elevation, in the opposite extreme of the ordination, coinciding with higher rock cover (Supporting Information Table S4; Figure 4).

**Figure 5:**
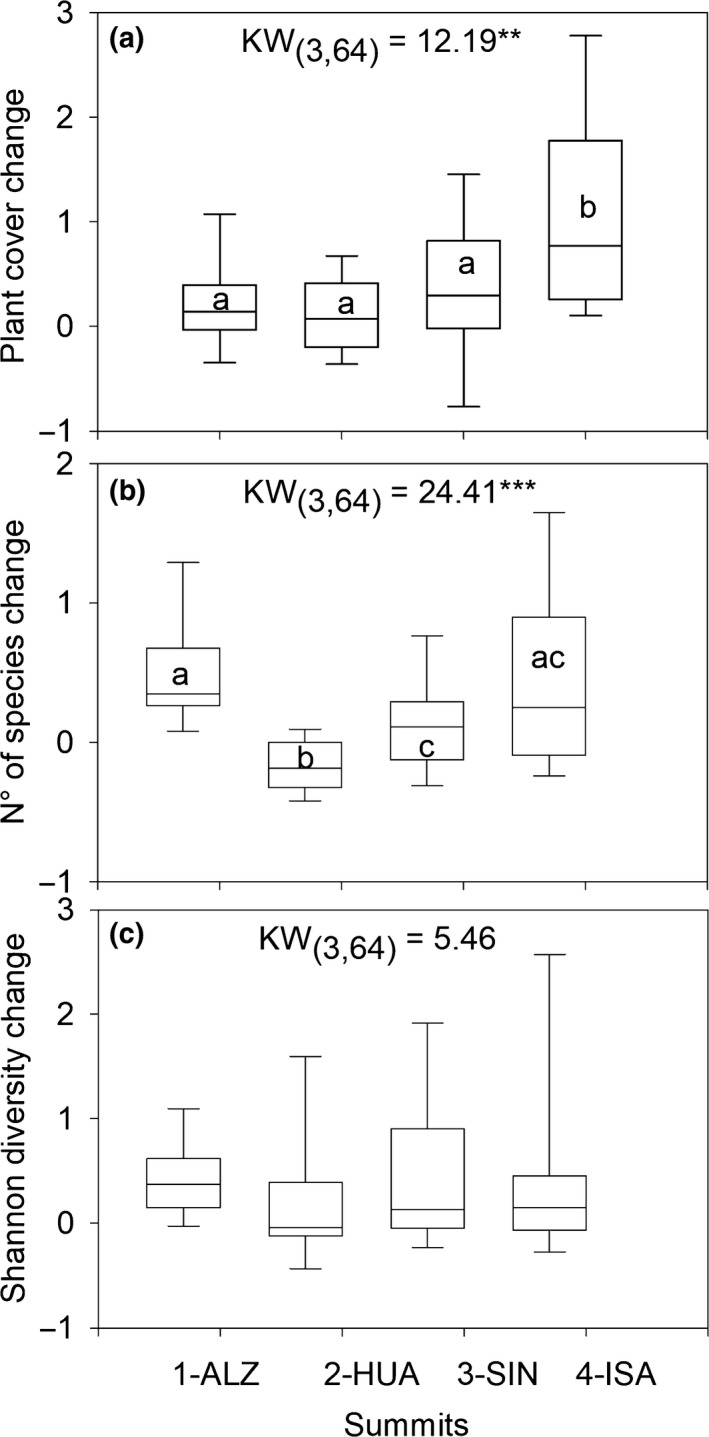
Box plot of relative change in (a) plant cover, (b) species richness, and (c) Shannon diversity index in the four summits in 11 years (Resurvey 2‐ baseline/baseline). **p < 0.001, ***p < 0.0001

After 9/11 years, relative change in plant cover was greatest in the highest summit, 4‐ISA where plant cover increased twofold (Figure 5a; Supporting Information Figure S3); this increase was driven by encroachment of cushion species that were already present in previous surveys (mainly Pycnophyllum convexum). The observed increase in plant cover was primarily concentrated on the western and southern aspects. However, overall plant cover on the highest summit was low (0.1%–3% total cover per plot), so relatively small increases in cover resulted in a high proportional difference (Figure 5a). The lowest summit 1‐ALZ showed the greatest gain in species richness (11 to 16 species per m^2^), significantly different than 2‐HUA summit, where species richness declined in the period (11 to 9 species per m^2^; Table 1, Figure 5b). The relative change in species diversity was not significantly different between summits (Figure 5c). Species turnover was highest in the lower summit 1‐ALZ, with 49% turnover (KW = 9.7, *p* = 0.02) and in northern and western slopes (47%; KW = 8.8, *p* = 0.03; Table 1).

#### 3.2.5 Distribution and dynamic of life forms

Life forms changed along the temperature gradient, within aspects and through time. The dominant life forms in terms of percentage cover (%), across summits and censuses were cushion plants (7%; higher in 2‐HUA with 10%) and tussock grasses (5.3%; higher in lower summits with 9.5%), followed by non‐tussock grasses (2%), shrubs (1.6%; predominating in 1‐ALZ with 3%), rosettes (1.6%), prostrate herbs (1.2%), erect herbs (0.6%) and ferns (0.02). All life forms were present in the four summits except tussock grasses which were absent in 4‐ISA. Cushions and shrubs were dominant in northern aspects (12.3% and 3.8%, respectively), tussock grasses, in southern and western (7.8%) and non‐tussock grasses in eastern aspects (3%).

Across all summits, non‐tussock grasses, erect herbs, rosettes, and the average of total life forms increased significantly over time (*p* < 0.05; Supporting Information Figure S4). Non‐tussock grasses increased significantly in all summits, mainly in northern aspect, while erects and rosettes increased in the lowest summit, where major changes occurred (with increases of 6.9% of life form cover). In this summit, the increase was mainly due to extant and new species of rosettes (e.g., Gomphrena meyeniana, Hypocheris eremophylla, Geranium sessiliflorum), erect herbs (Olsynium junceum and Calceolaria glacialis), and non‐tussock grasses (e.g., Poa kurtzii and Nasella rupestris). Other observed changes were not significant; 2‐HUA, tussock grasses tended to increase (by 2.5%), in 3‐SIN, shrubs (from the eastern aspect, particularly) tended to decrease (3.6% of decrease), and in 4‐ISA cushion plants tended to increase by 2% (e.g., P. convexum; Supporting Information Figure S4).

## 4 DISCUSSION

We found that temperature, particularly mean and minimum temperatures, and topographic features such as aspect, which modify the thermal conditions, were the primary environmental determinants of species richness, composition, and plant cover at the plot scale. Our findings agree with previous works in which temperature and sun exposure are proposed as major environmental constraints in high mountain ecosystems (Cuesta et al., 2017; Fernández Calzado, Molero Mesa, Merzouki, & Casares Porcel, 2012; Halloy, 1989; Kazakis, Ghosn, Vogiatzakis, & Papanastasis, 2006).

Cumbres Calchaquíes is characterized by high diversity and richness compared to other Andean sites (Cuesta et al., 2012). We recorded a total of 139 species in the four summits (2.2 ha), corresponding to 69% of the species recorded for the whole area by Halloy (2002; 201 species in 15,000 ha, above 4,000 m a.s.l., including wetlands and areas outside of summits). A total of 217 species are now listed for the area including 16 species not recorded in 2002. From the 14 new species reported in this study, six are probably new for the area, recorded at higher elevation than literature (e.g., Hypoxis decumbens, Sisyrinchium hypsophilum) and had been observed at lower elevation close to the area (e.g., Stevia chamedrensys), most of them are perennial herbs widely distributed in the Andes (Raimúndez & Ramirez, 1998), suggesting some upward movements, probably in response to short‐term climate variability, instead of long‐term trends, both important for modeling vegetation communities (Anthelme et al., 2017). In this study, vegetation variables were analyzed at a small spatial scale (1m^2^ plot) which captured 78% (109 species) of the total richness recorded in the four summits (Table 1). We found higher species richness than other puna (Seasonal high elevation grasslands) sites, with similar plant cover, e.g., Apolobamba and Sajama in Bolivia (64 and 48 species, respectively), but similar to paramo (non‐seasonal high elevation grasslands) sites e.g., Parque Nacional Podocarpus in Ecuador and Pacaipampa in Peru (84 and 82 species, respectively; Cuesta et al., 2012), where plant cover percentage is higher (Cuesta et al., 2017). Species richness does not follow the same temporal pattern at the summit and plot scales (neither plant life form); at the summit scale, species richness decreased in the 11 years over the four summits, while at the plot scale, it increased over time in summits 1 and 3. Thus, both scales are necessary to interpret dynamics in species composition; the plot smaller scale is more reliable for temporal analysis in cover terms, because it is more accurate and conservative in detecting changes, and reflects the high turnover rate of the plant community, while, the summit broader scale is better focused on the summit species pool for interpreting changes in species richness (Pauli et al., 2015; Zimmer et al, 2014).

The environmental gradient is also reflected in aspect and can be comparable to the elevation gradient in terms of temperature properties determining vegetation community (Sklenář et al., 2016). The highest temperature and diurnal thermal amplitude in the northern aspects, associated with high species richness, diversity and high community turnover over time, may promote the coexistence of species with wider temperature range requirements, mainly species adapted to warmer diurnal temperature, or the effect of degree days on phenology and growth (Oberbauer et al., 2013). Some of these plants, particularly species in the highest summits, may be frost‐tolerant species that resist high thermal amplitudes (Sierra‐Almeida, Cavieres, & Bravo, 2009; Squeo et al., 1996), such as rosettes (e.g., Draba sp), cushion plants (e.g., Pycnophyllum convexum), and shrubs (e.g., Tetraglochin cristatum). As many climate models predict increases of frost and drought events (Easterling, Meehl, Parmesan, Changnon, & Karl, 2000), frost‐tolerant species are particularly important. Eastern aspects showed comparable patterns to northern ones in terms of temperatures and vegetation distribution; whereas southern aspects, the coldest one, with the lowest diurnal thermal amplitude, were characterized by lower diversity and plant cover (Tables 1 and 2). The western aspects, also presented low minimum and mean temperature, but higher species richness than the southern aspects, which may reflect the influence of higher cloud cover in the afternoon and wind, which comes predominantly from the west, particularly in winter, with lots of easterlies in summer, particularly on the three highest summits (Halloy pers. com, Korner 2012). These results support the hypothesis that temperature, differing with elevation and aspect determine vegetation composition and distribution through differences in insolation period. These differences are reflected in several variables that affect vegetation communities, such as light intensity, soil temperature and moisture, and length of the growing season (Kutiel & Lavee, 1999; Maren, Karki, Prajapati, Yadav, & Shrestha, 2015).

Temperature was the main environmental filter separating species in ordination space, and it was associated with plant and rock cover, in different ways (and with elevation; Figure 4). In general, there was no clear temporal trajectory of vegetation in plots, but in most cases plots of second resurvey tended to assimilate to the baseline in the ordination space. The net trajectory between baseline and second resurvey (not shown), indicates minor changes, mainly in lower summits. This may highlight that in these mountains and at this temporal scale, vegetation responds to short‐term environmental variability, reflected in temperature and precipitation (Supporting Information Figure S1), and probably the distance index (Bray Curtis) is more sensitive to poorer and less greenery surfaces, reflected in longer trajectories (Figure 4). Highest summits are more heterogeneous within aspects of the same summits, reflected in the ordination space (and in the higher coefficient of correlation, not shown), for example, 4‐ISA east, the warmest slopes, combines characteristics from high elevation such as vegetation composition with high plant cover similar to lower summits (16% average in the three censuses, more than the double related to the others aspects of 4‐ISA); taller vegetation dominated by cushion plants and shrubs (e.g., Pycnophyllum convexum and Mulinum axiliflorum), which may generates an optimum microclimate (Korner 2012), functioning as nurse species, facilitating the establishment of other species, particularly observed in loose cushion plants such as the genus Pycnophyllum (Anthelme, Cavieres, & Dangles, 2014). 4‐ISA west in the extreme of axis 2, presented the lowest number of species and cover (9 species and 1.4% plant cover), close to 4‐ISA north with high rock and gravel cover, low bare soil, and low plant cover, probably, reflecting the substrate characteristic and the microclimate conditions generated by characteristics of the topography (Graae et al., 2018). 3‐SIN east, similar than 4‐ISA east, presented higher plant cover than other aspects of the summit, e.g. shrubs, cushion plants and also higher organic matter cover. The highest summit is characterized by few typical plant species, such as Aschersionodoxa cachensis, Arenaria rivularis, Valeriana pycnantha. In other mountains, these species are associated to crioturbed soils and rocky areas (Cano et al., 2011), thus, being highly vulnerable to global warming, so they can be useful as indicator species. Our study, as many others (aforementioned), focused its efforts in trying to understand vegetation community dynamics by inferring their climate‐driven distributions, to predict future communities trajectories in pursuit of its conservation, however, prediction may be a real challenge linked to novel climates (more vulnerable in tropics and subtropics) that may promote “no‐analog communities”, that is, nonexistent communities under current conditions (Williams & Jackson, 2007).

Our results suggest that vegetation dynamics (mainly a general increase in diversity and promotion of non‐tussock grasses and other small herbs) may be responding to short‐term variability in temperature and humidity combined with local factors (from different sources) in each summit. The low plant cover at the highest summit seemed to respond quite quickly to temperature changes (doubling plant cover). At the lowest summit, an increase in the three vegetation metrics is observed, as in other mountains in the world (Gottfried et al., 2012; Venn, Pickering, & Green, 2014), with abrupt changes in species richness in the first 5 years, probably responding to the short‐term variability in temperature and humidity combined with anthropogenic variables, such as grazing or tourism (Steinbauer et al., 2018; Yager et al., 2008). Some studies evidenced that decreasing in domestic cattle, driven by changes in land use (promoted by socioeconomic factors; e.g., mining and tourism) may favor native herbivores populations (Barros, Monz, & Pickering, 2015; Izquierdo et al., In press; Steinbauer et al., 2018). This grazing transition may affect vegetation communities in the entire elevation gradient, a hypothesis to be tested in the future. At the second summit, 2‐HUA, vegetation changes (decrease in species richness and increase in plant cover) may be driven by the combination of climate variability and local biological factors such as competition, for example; tussock grasses may be limiting plant colonization, by competing for local resources (Halloy, 1985a). The particular hypsography of these mountains, with a plateau at middle elevation, may also influence the linear relationship expected between elevation and species richness, observed at summit scale (Elsen & Tingley, 2015). In 3‐SIN, changes in vegetation seems to respond to the climate variability combined with local abiotic effects, such as sun exposition and wind, having an effect over predominant plant cover (some shrubs located in the east aspect dried up), but after the 10 years of analysis a recovery of the three vegetation variables is observed (Table 1; Supporting Information Figure S1). Diversity index increases significantly over time across the four summits, reflecting the short‐term temperature and precipitation variability, as well (lower diversity at the baseline coincided with dry and warm period; Supporting Information Figure S1), and as it encompasses both variables, species cover and richness, could be a good indicator of vegetation community dynamics.

The area suffered a period of drought since 1980, evidenced in a general decrease of lake levels (Halloy, 2002, Casagranda, 2010), until 2012, when lakes reached the highest levels recorded in recent years (Carilla, unpubl.). During the period 2008 – 2012, the area was influenced by the coupled oceanic ‐ atmospheric event, El Niño Southern Oscillation (ENSO) ‐ La Niña phase, manifested by higher precipitation at this elevation (Supporting Information Supporting Information Figure S1), like other subtropical and tropical high Andean areas (Carilla, Grau, Paolini, & Morales, 2013; Morales et al., 2015). On the contrary, El Niño phase is linked to dry periods at these elevations (e.g., El Niño 1997–98, 2003–04, 2015–2016; Morales et al., 2015). ENSO oscillations are reflected in lake levels recorded off and on since 1926 (Halloy, 1985a, 2002; Minetti, 2015). SOI (up to June) was in El Niño phase before the 2007 baseline, in contrast, the period 2008 to 2012 (resurvey 1) was in La Niña phase; and 2013 to 2017 tended again to El Niño; https://www.bom.gov.au/climate/current/soihtm1.shtml). The 2008 – 2012 humid period in Huaca Huasi corresponds to a slowing down of the global trend in temperature increase, evidenced in other tropical areas (Clement & DiNezio, 2014). These inter‐annual variability cycles may help explain temperatures, precipitation, lake levels, and hence vegetation variations over that period, observed in other Andean systems (Carilla et al., 2013).

There are worries that plant community composition and perhaps community functioning will be negatively affected by global change in the future. Our study showed that over an 11‐year period, total plant cover, species richness and diversity were positively associated with temperature. Some signals highlighted in this study suggest that temperature is changing at different temporal scales (e.g., Increase of 0.5°C in minimum temperature between 2009 and 2015) and vegetation dynamics may be responding to these changes. Although our data may be not enough to support the observed relationship, we suggest that trends in vegetation change respond to short‐term in temperature and precipitation variability, which is strongly influenced by ENSO, as in the central Andes (Christie et al., 2009; Morales et al., 2015), thus, it may take much longer than one decade for trends to became significant, due to the natural time lags in plant community responses to shifts in rainfall and temperature. This hypothesis should be tested with longer term monitoring studies; however, our data provide an important baseline with which to document changes in these sensitive alpine plant communities as global climate continues to change.

## 5 CONCLUSION

Soil temperature and topography (aspect and elevation) were the primary environmental determinants of vegetation dynamics at these spatial and temporal scales, with soil temperature decreasing significantly with elevation and showing a positive trend over the six years of records. Greater plant cover, species richness, and diversity tended to occur at the lower summits and in the warmer northern and eastern aspects, while the lower species richness and diversity occurred in the highest summit and in the colder southern aspects. After 9/11 years, we observed that diversity index increased in the four summits, species richness increased in the lower summit and plant cover increased in the highest summit, with a general increase in non‐tussock grasses (at plot scale). Our results suggest that vegetation dynamics may be associated with short‐term variability of soil temperature and precipitation, influenced by ENSO at this elevation.

## CONFLICT OF INTEREST

None declared.

## AUTHORS CONTRIBUTION

JC, AG, SC, and SH conceived of and designed the study, they also collected data and conducted initial data analyses. SC identified species, JC, FC, and AM contributed to statistical analysis. JC wrote the first draft of the manuscript, all authors contributed substantially to revisions.

## DATA ACCESSIBILITY

Data available from the Dryad Digital Repository: https://doi.org/10.5061/dryad.3gk6pr4
