# Supplementary material for: Vegetation trends over eleven years on mountain summits in NW Argentina
Source: Ecol Evol. 2018 Nov 14;8(23):11554–67. doi: 10.1002/ece3.4602 (PMC6303700; doi:10.1002/ece3.4602)
Supplement: Supplementary file 1 [file ECE3-8-11554-s001.docx]

**Supporting information**

Data S1. New and missing species at summit scale

We recorded 125 species in the baseline survey, 131 species in resurvey 1 and 114 in resurvey 2, a total of 139 species in the three censuses. In resurveys 1 and 2, 14 species were newly recorded, and 13 species were no longer recorded (for the calculation of new and missing species we did not consider Poaceae). One species was exclusively recorded in the baseline (*Werneria pygmaea*), two species exclusively in resurvey 1 (*Chersodoma antennaria* and *Stevia chamaedrys*), and only one species recorded exclusively in resurvey 2 (*Carex maritima,* but had been recorded by Halloy 1985). Two species were not seen in resurvey 1 but in the baseline and resurvey 2 (*Gomphrena umbellata* and *Chenopodium carnosulum)*. Ten species were recorded at higher elevation than in literature, from these, three were new in resurveys 1 and 2; *Hypoxis decumbens, Mastigostyla mirabilis* and *Sisyrinchium hypsophilum*. The number of new species arriving from the baseline to resurvey 2 decreased with altitude. The lowest summit 1-ALZ showed the highest number of new species (14 species), most of them on the east and south facing slope. The half of the 14 new species of 1-ALZ had already been recorded in the baseline on at least one other summit, while the remaining seven species were not recorded on any of the four summits before. The number of species not found again was higher on the lower summit (17), and in the north facing slopes (26). On the highest summit 4-ISA, the number of missing species (15) were higher than the number of new species (5). Of the 35 botanical families recorded, three (Alstroemeriaceae, Crassulaceae and Rubiaceae) were not recorded in resurvey 2, while one (Cyperaceae) appeared in resurvey 2 (Table 1. Appendix 1).

**Supporting tables**

**Table S1**. Linear regression between elevation and soil monthly mean temperature (2009 – 2012 period)

*** 0.001 ** 0.01 * 0.05

| **Elevation** | Estimate | Std Error | T value | Prob (>\|t\|) |  |
| --- | --- | --- | --- | --- | --- |
|  |  |  |  |  |  |
| (Intercept) | 23.478 | 5.178 | 4.534 | 0.045 | * |
| Minimum average temperature | -0.006 | 0.001 | -4.750 | 0.042 | * |
| Adjusted R^2^ 0.92 |  |  |  |  |  |
| (Intercept) | 29.671 | 4.405 | 6.736 | 0.0213 | * |
| Mean temperature | -0.005 | 0.001 | -5.326 | 0.033 | * |
| Adjusted R^2^ 0.93 |  |  |  |  |  |
| (Intercept) | 31.985 | 6.353 | 5.034 | 0.037 | * |
| Maximum average temperature | -0.004 | 0.0014 | -2.508 | 0.129 |  |
| Adjusted R^2^ 0.63 |  |  |  |  |  |

**Table S2**. Plant cover, species richness and Shannon-wiener diversity index per aspect (average of the four 1m^2^ plots) within summits in the three censuses.

|  | Plant cover | | | Species richness | | | Shannon diversity index | | |
| --- | --- | --- | --- | --- | --- | --- | --- | --- | --- |
| **ALZ** | BL | RM1 | RM2 | BL | RM1 | RM2 | BL | RM1 | RM2 |
| E | 27.8 | 26.5 | 26.9 | 11.3 | 14.0 | 15 | 1.88 | 2.27 | 2.53 |
| N | 29.1 | 36.8 | 37.7 | 10.3 | 17.5 | 17.3 | 2.50 | 3.02 | 3.16 |
| S | 27.95 | 28.5 | 26.0 | 11.3 | 12.0 | 13.3 | 1.64 | 1.75 | 1.97 |
| W | 25.2 | 37.1 | 32.8 | 12.3 | 18.5 | 20.0 | 1.77 | 2.72 | 3.15 |
| **HUA** |  |  |  |  |  |  |  |  |  |
| E | 17.6 | 9.2 | 17.1 | 9.5 | 9.0 | 7.3 | 1.6 | 2.36 | 1.94 |
| N | 28.4 | 25.2 | 37.0 | 13.5 | 11.3 | 12.8 | 2.44 | 2.5 | 2.38 |
| S | 26.9 | 28.7 | 33.1 | 8.5 | 7.5 | 7.5 | 1.24 | 1.10 | 1.04 |
| W | 30.5 | 34.5 | 24.2 | 12.8 | 14.3 | 10 | 1.86 | 1.85 | 1.83 |
| **SIN** |  |  |  |  |  |  |  |  |  |
| E | 41.3 | 25.5 | 27.1 | 14.5 | 15.3 | 15 | 1.65 | 1.83 | 2.26 |
| N | 5.1 | 5.5 | 8.6 | 6.8 | 5.0 | 6.3 | 1.04 | 1.17 | 1.44 |
| S | 6.0 | 6.2 | 7.9 | 4.3 | 3.8 | 5 | 1.08 | 0.66 | 1.12 |
| W | 7.6 | 8.8 | 11.2 | 7.0 | 7.3 | 9.3 | 1.74 | 1.36 | 1.93 |
| **ISA** |  |  |  |  |  |  |  |  |  |
| E | 13.3 | 16.2 | 20.5 | 6.8 | 7.8 | 9.5 | 1.58 | 1.7 | 1.6 |
| N | 3.0 | 2.8 | 4.9 | 6.0 | 5.8 | 8 | 1.81 | 1.94 | 2.16 |
| S | 5.1 | 7.0 | 8.2 | 5.0 | 4.0 | 5.3 | 1.27 | 1.03 | 1.35 |
| W | 1.0 | 1.12 | 2.1 | 2.8 | 3.8 | 4.5 | 0.90 | 1.51 | 1.63 |

**Table S3.** Linear mixed model (LMM) and Generalized linear mixed models (GLMM) output, for differences in plant cover, species richness and Shannon wiener diversity index within aspects and censuses (fixed effects), between summits (random effect).

| Plant cover (Gaussian distribution, root square transformation; Linear mixed model) | | | | | | | | | | | | | | |  |
| --- | --- | --- | --- | --- | --- | --- | --- | --- | --- | --- | --- | --- | --- | --- | --- |
|  |  | |  | | |  | | |  | |  | | |  |  |
| Random effects | | |  | | |  | | |  | |  | | |  |  |
| Groups | Name | | Variance | | | Std.Dev. | | |  | |  | | |  |  |
| **Summit** | (Intercept) | | 1.976 | | | 1.406 | | | **<0.0001** | |  | | |  |  |
| Residual | 1.647 | | 1.283 | | |  | | |  | |  | | |  |  |
| Fixed effects: | | | | | | | | | | | | | | |  |
|  | Sum Sq | | Mean Sq | | | Num DF | | | DenDF | | F.value | | | Pr(>F) |  |
| Aspect | 22.1253 | | 7.3751 | | | 3 | | | 183 | | 4.4775 | | | **0.0046** |  |
| Censuses | 3.5504 | | 1.7752 | | | 2 | | | 183 | | 1.0777 | | | 0.3425 |  |
|  |  | |  | | |  | | |  | |  | | |  |  |
| Species richness (Poison distribution; Generalized linear mixed model) | | | | | | | | | | | | | | |  |
| Variance Components for Random Effects (P-values are one-tailed): | | | | | | | | | | | | | |  |  |
|  | Estimate | | Std.E | | | z Value | | | Pr(>\|z\|)/2 | |  | | |  |  |
| Summit | 0.11147 | | 0.08109 | | | 1.375 | | | **0.0846** | | . | | |  |  |
| Fixed Effects: | | | | | | | | | | | | | | |  |
|  | | Estimate | Std. E | | | z value | | | Pr(>\|z\|) | |  | | |  |  |
| Aspect E | | 2.32993 | 0.10651 | | | 21.875 | | | **2.00E-16** | | *** | | |  |  |
| Aspect N | | 2.21609 | 0.10756 | | | 20.604 | | | **2.00E-16** | | *** | | |  |  |
| Aspect S | | 1.89529 | 0.11115 | | | 17.051 | | | **2.00E-16** | | *** | | |  |  |
| Aspect W | | 2.23258 | 0.1074 | | | 20.788 | | | **2.00E-16** | | *** | | |  |  |
| Resurvey 1 | | 0.09547 | 0.05792 | | | 1.648 | | | **0.0993** | | . | | |  |  |
| Resurvey 2 | | 0.15289 | 0.05715 | | | 2.675 | | | **0.00746** | | ** | | |  |  |
|  | |  |  | | |  | | |  | |  | | |  |  |
| Shannon Wiener diversity index (Gaussian distribution; linear mixed model) | | | | | | | | | | | | | | | |
|  | |  | |  |  | | |  | | | |  |  | | |
| Random effects: | | | |  |  | | |  | | | |  |  | | |
| Groups | | Name | | Variance | Std.Dev. | | |  | | | |  |  | | |
| Summit | | (Intercept) | | 0.02498 | 0.1581 | | | **< 0.0001** | | | | *** |  | | |
| Residual | | 0.06379 | | 0.2526 |  | | |  | | | |  |  | | |
| Fixed effects | | | | | | | | | | | | | | | |
|  | | Sum Sq | | Mean Sq | | | Num DF | Den DF | | F.value | | | Pr(>F) | | |
| Aspects | | 3.4537 | | 1.1512 | | | 3 | 183 | | 18.0464 | | | <1e-07 | | |
| censuses | | 0.5379 | | 0.2689 | | | 2 | 183 | | 4.2158 | | | 0.0162 | | |
|  | |  | |  | | |  |  | |  | | |  | | |
| Signif. codes: 0 ‘***’ 0.001 ‘**’ 0.01 ‘*’ 0.05 ‘.’ 0.1 ‘ ’ 1 | | | | | | | | | | | | | | | |
|  | |  | |  |  | | |  | |  | | |  | | |

Table S4. Kendal Correlations between both axes of NMDS and main matrix (species) and secondary matrix. Bonferoni adjustment: p<0.0005 and p<0.003, respectively (significant correlations with bold)

| Species | Axis 1 | Axis 2 |
| --- | --- | --- |
| Adesmia schickendantzii | **0.526** | **-0.356** |
| Anatherostipa mucronata | 0.085 | **0.363** |
| Arenaria rivularis | **-0.375** | -0.031 |
| Aschersoniodoxa cachensis | **-0.349** | 0.013 |
| Astragalus bustillosi | **0.41** | -0.207 |
| Astragalus peruvianus | **0.314** | **-0.523** |
| Azorela compacta | **0.354** | -0.018 |
| Brayopsis monimocalix | -0.053 | **0.382** |
| Calceolaria glacialis | **0.345** | -0.016 |
| Cardionema burkartii | **0.552** | **-0.374** |
| Cerastium tucumanense | **0.442** | -0.259 |
| Deyeuxia sp | **0.45** | **-0.441** |
| Festuca sp | **-0.392** | 0.004 |
| Gamochaeta erythractis | **0.485** | -0.299 |
| Geranium sessiliflorum | **0.465** | **-0.414** |
| Gomphrena meyeniana | **0.543** | **-0.337** |
| Hypochaeris eremophila | **0.424** | **-0.407** |
| Jarava leptostachya | **0.456** | -0.294 |
| Lepidium meyeni | 0.041 | **-0.447** |
| Luciliocline burkartii | **0.472** | **-0.416** |
| Mulinum axiliflorum | -0.09 | **0.611** |
| Nassella rupestris | **0.624** | **-0.508** |
| Paronichia hieronimi | **0.444** | -0.297 |
| Parastrephia lucida | 0.212 | **0.361** |
| Plantago sericea var. sericea | **0.551** | **-0.556** |
| sisyrinchium hypsophilum | **0.399** | -0.213 |
| Sisyrinchium junceum | **0.544** | -0.34 |
| Tetraglochin cristatum | **0.472** | -0.341 |
| Tetraglochin inerme | -0.005 | **-0.366** |
| Valeriana pycnantha | **-0.495** | 0.051 |

Secondary Matrix

| Variables | Axis 1 | Axis 2 |
| --- | --- | --- |
| Mean temperature | **0.63** | **-0.34** |
| Min average temperature | **0.54** | **-0.55** |
| Max average temperature | **0.39** | -0.2 |
| Mean temp growing season | **0.54** | **-0.39** |
| Mean temp non growing season | 0.27 | **-0.56** |
| Total plant cover | **0.54** | **-0.32** |
| Cushion plants cover | **0.38** | -0.03 |
| Non tussock grasses cover | **0.32** | **-0.46** |
| Prostrate cover | **0.51** | **-0.39** |
| Rosette cover | **0.33** | **-0.63** |
| Shrubs cover | **0.32** | 0.156 |
| Tussock grasses cover | 0.24 | **-0.35** |
| Bare soil cover | 0.28 | **-0.32** |
| Organic matter cover | **0.35** | -0.24 |
| Rock cover | **-0.41** | **0.35** |

Supporting figures


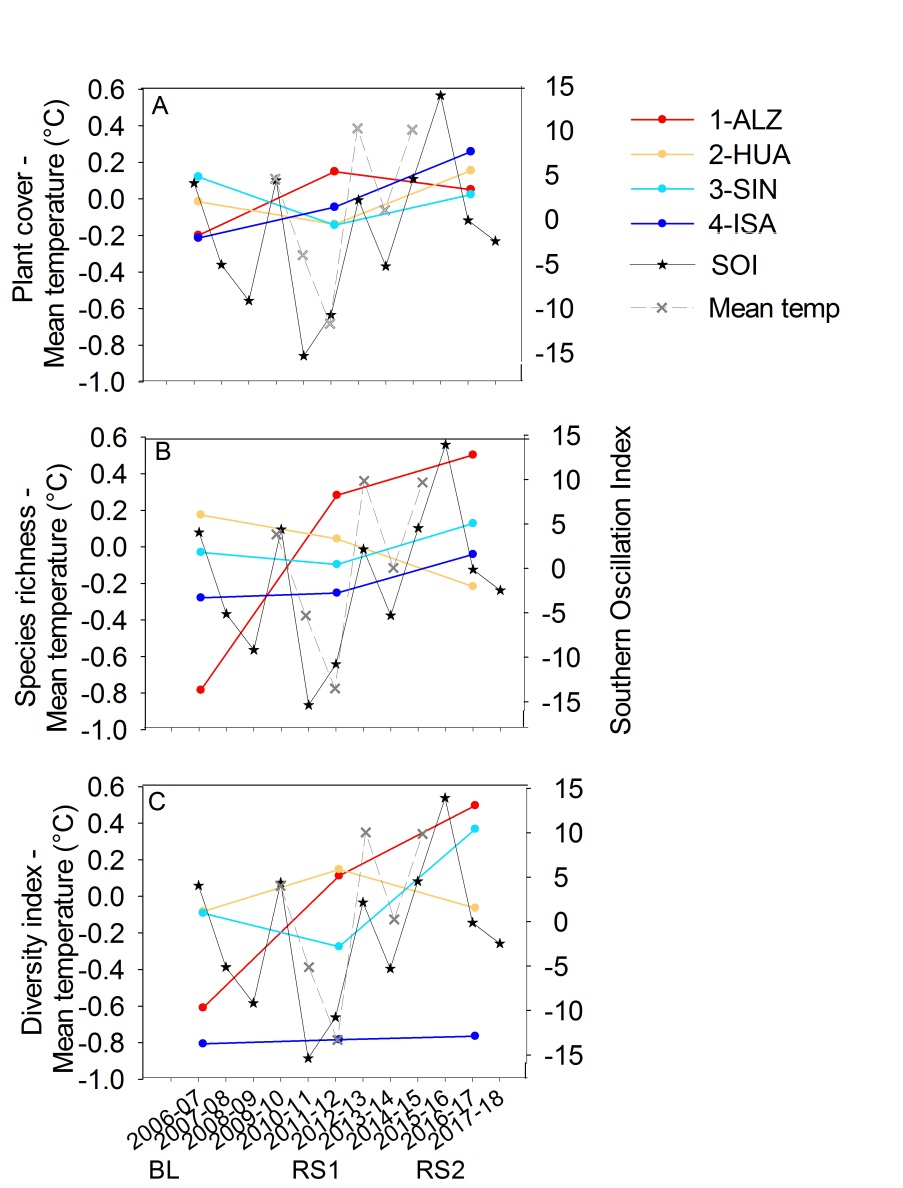


**Figure S1.** Anomalies of plant cover (A), species richness in 1 m^2^ (B) and Shannon diversity index (C) in the three censuses (X-axis) compared to mean temperature anomaly (°C; Y-axis left) and Southern Oscillation index (SOI; Y-axis right). BL: baseline, Resurvey 1: RS1, Resurvey 2: RS2. SOI is shown inversed to facilitate the comparison with temperature.


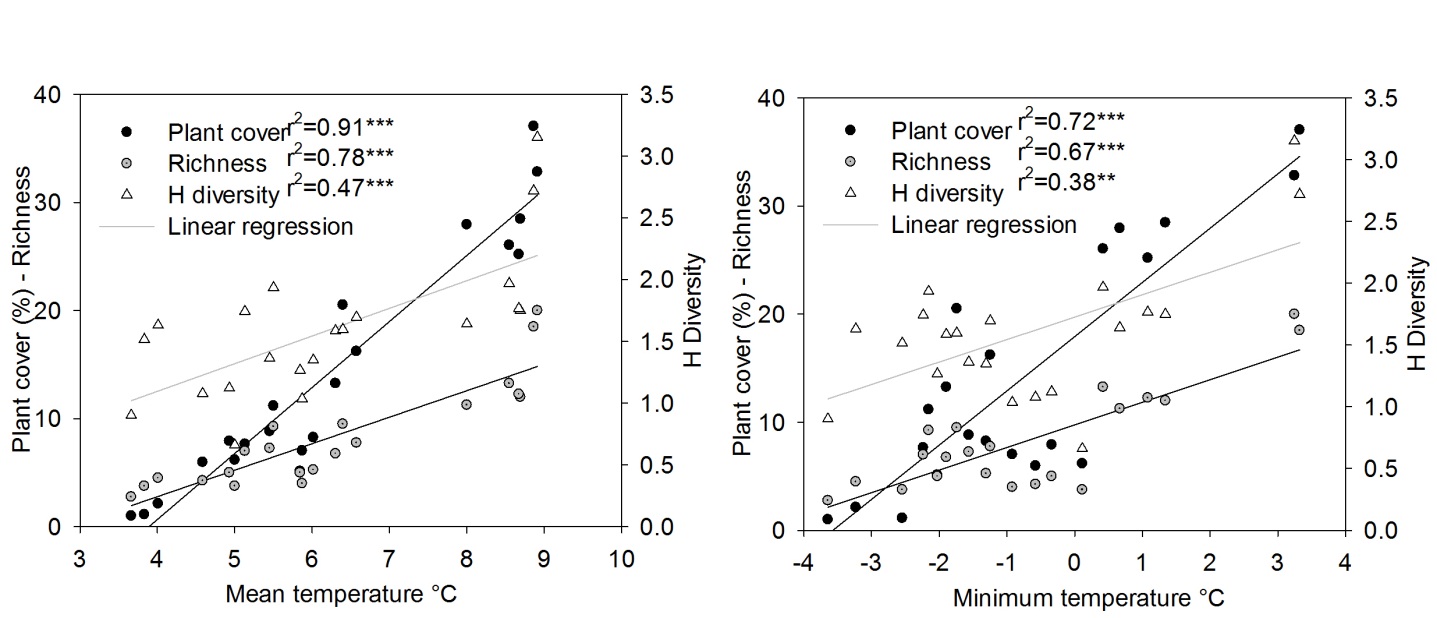


**Figure S2.** Linear regressions between plant cover (black circle), species richness (grey circle) and Shannon diversity H (white triangle), with mean and minimum temperature of the seven plots with longer temperature records (2009-2015; N= 21: seven sites by three censuses). R^2^ is indicated for each variable **p<0.001, ***p<0.0001


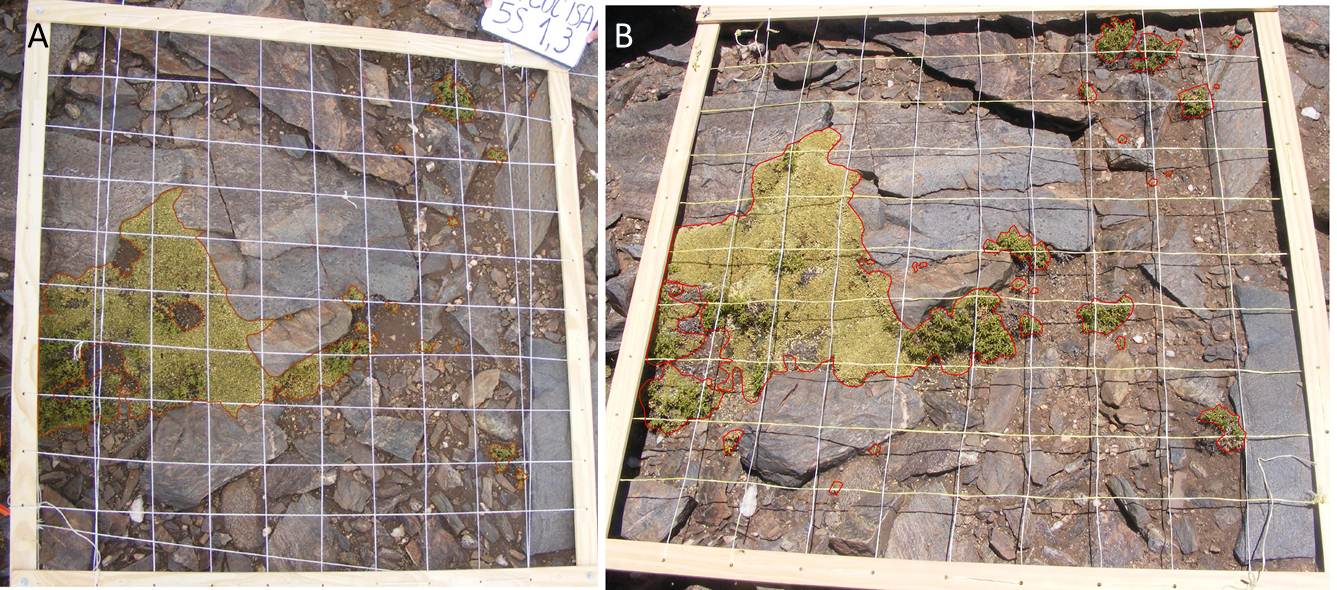


Figure S3. Example of a southern slope plot of the highest summit where plant cover increased over time, A) baseline, B) resurvey 2.


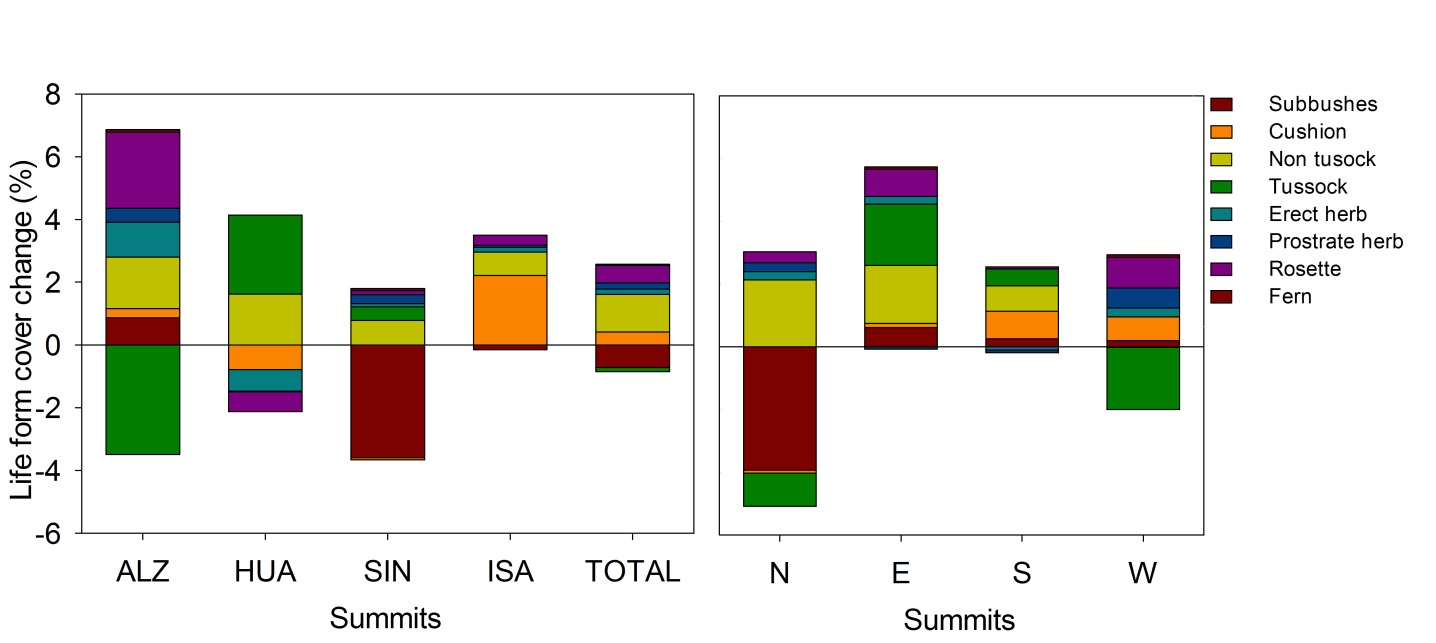


**Figure S4**. Changes in plant life forms cover (2006/08-2017) in summits and aspects.
