# Supplementary material for: Vegetation trends over eleven years on mountain summits in NW Argentina
Source: Ecol Evol. 2018 Nov 14;8(23):11554–67. doi: 10.1002/ece3.4602 (PMC6303700; doi:10.1002/ece3.4602)
Supplement: Supplementary file 2 [file ECE3-8-11554-s002.docx]

Appendix 1. Species list ordered by botanical family, summit recorded, life forms, census: base line (BL), re-survey 1 (RS1), re-survey 2 (RS2), and some observations. General distribution and elevational range based on the Darwinion Botanic Institute (<http://www.darwin.edu.ar/Proyectos/FloraArgentina/fa.htm>), and TROPICOS (<http://www.tropicos.org>). Ar: Argentina, Bo: Bolivia, Ch: Chile, Pe: Peru, Br: Brazil, Ur: Uruguay, Par: Paraguay, NWA: northwestern Argentina.

| **Scientific name** | **Summit** | **Life form** | **Census** | **Observation** | **General distribution** | **Elevational range (m asl)** |
| --- | --- | --- | --- | --- | --- | --- |
| **Alstroemeriaceae** |  |  |  |  |  |  |
| *Alstroemeria pygmaea* | 1 | Erect herb | BL, RS1 |  | Ar, Bo | 3000-4400 |
| **Amaranthaceae** |  |  |  |  |  |  |
| *Gomphrena meyeniana* | 1, 3 | Rosette | BL, RS1, RS2 |  | Ar, Bo, Ch | 2000-4500 |
| *Gomphrena umbellata* | 1 | Rosette | BL, RS2 |  | Ar, Bo, Ch | 3000-4000 |
| **Apiaceae** |  |  |  |  |  |  |
| *Azorella compacta* | 1, 3 | Cushion plant | BL, RS1, RS2 |  | Ar, Bo, Ch | 3500-5200 |
| *Bowlesia tropaeolifolia* | ALL | Erect herb | BL, RS1, RS2 |  | Ar, Bo, Ch | 100-4500 |
| *Mulinum axilliflorum* | ALL | Sub-bush | BL, RS1, RS2 |  | Endemic NWA | 4000-4500 |
| **Asteraceae** |  |  |  |  |  |  |
| *Chersodoma antennaria* | 1 | Erect herb | RS1 | High probability of being new. Halloy (1985a) recorded Senecio diclina |  |  |
| *Conyza deserticola* | 1, 3 | Erect herb | BL, RS1 |  | Ar, Bo, Ch | 2000-4400 |
| *Gamochaeta erythractis* | 1, 2, 3 | Prostrate herb | BL, RS1, RS2 |  | Endemic NWA | 2500-4000 |
| *Hieracium mandonii* | 1, 3 | Rosette | BL, RS1, RS2 |  | Ar, Bo | 2000-4000 |
| *Hypochaeris eremophila* | ALL | Rosette | BL, RS1, RS2 |  | Ar, Ch | 2800-4700 |
| *Hysterionica pulchella* | ALL | Erect herb | BL, RS1, RS2 |  | Endemic NWA | 2500-4000 |
| *Leucheria pteropogon* | 4 | Erect herb | RS1, RS2 | Possibly not seen among rocks at BL. Recorded by Halloy (1985a) in the vicinity of this summit | Ar, Ch | 3000-5100 |
| *Luciliocline burkartii* | ALL | Prostrate herb | BL, RS1, RS2 |  | Ar, Bo | 3000-4000 |
| *Mniodes schultzii* | ALL | Erect herb | BL, RS1, RS2 |  | Ar, Bo, Ch | 4000-5000 |
| *Mniodes santanica* | 1, 2, 3 | Erect herb | BL, RS1, RS2 |  | Ar, Bo | 4200-4400 |
| *Nassauvia axillaris* | 1, 3 | Sub-bush | BL, RS1, RS2 |  | Ar, Bo, Ch | 1000-5000 |
| *Oriastrum pulvinatum* | 3, 4 | Prostrate herb | BL, RS1, RS2 |  | Ar, Ch | 3000-4500 |
| *Oriastrum revolutum* | 1, 2 | Erect herb | BL, RS1, RS2 |  | Ar, Ch | 3100-4200 |
| *Oriastrum stuebelii var. abbreviatum* | ALL | Erect herb | BL, RS1, RS2 |  | Endemic NWA | 3000-4500 |
| *Parastrephia lucida* | 3 | Shrub | BL, RS1, RS2 |  | Endemic NWA | 2900-5000 |
| *Perezia ciliosa* | ALL | Rosette | BL, RS1, RS2 |  | Ar, Bo, Ch | 2500-4600 |
| *Perezia multiflora* | 1, 2 | Erect herb | BL, RS1, RS2 |  | Ar, Ch | 1000-4500 |
| *Perezia pungens* | ALL | Rosette | BL, RS1, RS2 |  | Ar, Bo, Ch | 3000-5000 |
| *Pseudognaphalium lacteum* | 1, 2, 3 | Prostrate herb | BL, RS1, RS2 |  | Ar, Bo | 3000-5000 |
| *Senecio adenophyllus* | 1, 2, 3 | Erect herb | BL, RS1, RS2 |  | Ar, Bo, Ch | 4000-5000 |
| *Senecio algens* | 4 | Erect herb | BL, RS1, RS2 |  | Ar, Bo, Ch | 4200-5200 |
| *Senecio anconquijae f. discoideus* | 1 | Erect herb | BL, RS1 | Found at higher altitude than literature | Endemic NWA | 3300-3400 |
| *Senecio asplenifolius* | 1, 4 | Erect herb | BL, RS1, RS2 |  | Endemic NWA | 3500-4500 |
| *Senecio candollei* | 2 | Rosette | BL, RS1, RS2 |  | Ar, Bo, Ch | 3300-5000 |
| *Senecio maculatus* | 1, 4 | Erect herb | BL, RS1, RS2 |  | Endemic NWA | 4000-4300 |
| *Senecio nutans* | 2, 3 | Erect herb | BL, RS1, RS2 |  | Ar, Bo, Ch | 3500-5000 |
| *Senecio* volckmannii | 4 | Cushion plant | BL, RS1 |  | Ar, Ch | 4300-5100 |
| *Stevia chamaedrys* | 1 | Erect herb | RS1 | High probability of being new. Observed at lower altitude. | Ar, Ch | 2000-4500 |
| *Trichocline reptans* | 1 | Rosette | BL, RS1, RS2 | Found at higher altitude than literature | Ar, Bo, Pa | 0-3300 |
| *Werneria cochlearis* | ALL | Rosette | BL, RS1, RS2 |  | Endemic NWA | 4000-4500 |
| *Werneria pygmaea* | 2, 3, 4 | Erect herb | BL | Link to moist soils, either was miss-identified in BL or had died out to drying conditions or could be overlooked when not in flower | Ar, Bo, Ch | 2500-5000 |
| **Boraginaceae** |  |  |  |  |  |  |
| *Phacelia nana* | ALL | Rosette | BL, RS1, RS2 |  | Ar, Bo, Ch | 0-4000 |
| *Phacelia secunda var. secunda* | 1, 2 | Prostrate herb | BL, RS1 |  | Ar, Bo, Ch | 2900-4400 |
| *Plagiobothrys congestus* | 1, 2, 3 | Rosette | RS1, RS2 | Link to moist soils. As it is fast growing could be colonizing during short wet summer. Probably present in BL but not recognized. Recorded by Halloy (1985a) | Endemic NWA | 2000-4500 |
| **Brassicaceae** |  |  |  |  |  |  |
| *Aschersoniodoxa cachensis* | 4 | Erect herb | BL, RS1, RS2 |  | Ar, Bo | 4000-5100 |
| *Brayopsis monimocalyx* | 1, 2, 3 | Rosette | BL, RS1, RS2 |  | Ar, Bo | 4000-4800 |
| *Descurainia depressa* | ALL | Erect herb | BL, RS1, RS2 |  | Ar, Bo, Ch | 3000-4600 |
| *Descurainia myriophylla* | 2, 3, 4 | Erect herb | BL, RS1, RS2 |  | Ar, Bo | 1900-4600 |
| *Draba macleanii* | 4 | Rosette | BL, RS1 |  | Endemic NWA | 3900-4900 |
| *Draba burkartiana* | 4 | Rosette | BL, RS1, RS2 |  | Endemic NWA | 4000-4500 |
| *Draba gilliesii* | 2, 3, 4 | Rosette | BL, RS1, RS2 |  | Ar, Ch | 400-4000 |
| *Lepidium meyenii* | ALL | Rosette | BL, RS1, RS2 |  | Ar, Bo, Ch | 2500-4500 |
| *Mancoa hispida* | 1, 2, 3 | Rosette | BL, RS1, RS2 |  | Ar, Bo, Ch | 3800-4800 |
| *Menonvillea virens* | 4 | Erect herb | BL, RS1, RS2 |  | Ar, Ch | 3000-4800 |
| *Parodiodoxa chionophila* | 3, 4 | Erect herb | BL, RS1, RS2 |  | Endemic NWA | 3500-5100 |
| *Physaria mendocina* | ALL | Rosette | BL, RS1, RS2 | Found at higher altitude than literature for Argentina | Ar | 200-3000 |
| **Cactaceae** |  |  |  |  |  |  |
| *Austrocylindropuntia verschaffeltii* | 1 | Cushion plant | BL, RS1, RS2 | Found at higher altitude than literature for Argentina | Ar, Bo | 1500-3000 |
| *Maihueniopsis boliviana* | 1, 2, 3 | Cushion plant | BL, RS1, RS2 |  | Ar | 3000-4500 |
| **Calceolariaceae** |  |  |  |  |  |  |
| *Calceolaria glacialis* | ALL | Erect herb | BL, RS1, RS2 |  | Ar, Bo | 3500-4100 |
| *Calceolaria santolinoides* | 2, 3 | Erect herb | BL, RS1 |  | Ar, Bo | 3400-4300 |
| **Calyceraceae** |  |  |  |  |  |  |
| *Calycera pulvinata* | ALL | Rosette | BL, RS1, RS2 |  | Andean dist., Ar to Ve. | 3600-4600 |
| *Moschopsis monocephala* | 1, 4 | Rosette | BL, RS1, RS2 |  | Ar, Ch | 4000-5000 |
| **Caryophyllaceae** |  |  |  |  |  |  |
| *Arenaria bisulca* | ALL | Erect herb | BL, RS1, RS2 |  | Ar, Pe | 1000-4500 |
| *Arenaria serpens* | 1, 2, 4 | Erect herb | RS1, RS2 | High probability of being new in RS1 | Ar, Bo, Ch, Pe | 500-4200 |
| *Arenaria pycnophylloides* | ALL | Prostrate herb | BL, RS1, RS2 | Found at higher altitude than literature | Endemic NWA | 3300-3800 |
| *Arenaria rivularis* | 4 | Erect herb | BL, RS1, RS2 |  | Ar, Ch | 3200-4700 |
| *Cardionema burkartii* | 1 | Prostrate herb | BL, RS1, RS2 |  | Ar, Bo | 1500-4600 |
| *Cerastium tucumanense* | ALL | Erect herb | BL, RS1, RS2 |  | Endemic NWA | 1000-4500 |
| *Paronychia cabrerae* | 1 | Prostrate herb | BL, RS1 | Possible confused with *P. hieronymi* in RS2 | Endemic NWA | 2800-4500 |
| *Paronychia hieronymi var. hieronymi* | 1 | Prostrate herb | RS1, RS2 | Recorded by Halloy (1985a) | Endemic NWA | 2500-4800 |
| *Pycnophyllum convexum* | ALL | Cushion plant | BL, RS1, RS2 |  | Endemic NWA | 4000-4500 |
| *Silene mandonii* | ALL | Rosette | BL, RS1, RS2 |  | Ar, Bo, Ch | 1500-4700 |
| **Chenopodiaceae** |  |  |  |  |  |  |
| *Chenopodium carnosulum* | 1 | Erect herb | BL, RS2 |  | Ar, Bo, Ch | 0-4200 |
| **Crassulaceae** |  |  |  |  |  |  |
| *Crassula connata var. connata* | 1, 2, 3 | Erect herb | BL, RS1 | Found at higher altitude than literature | Ar, Bo, Ch, Ur | 0-3300 |
| **Cyperaceae** |  |  |  |  |  |  |
| Carex maritima | 1 | Erect herb | RS2 | Recorded by Halloy (1985a) | Ar, Ch | 200-4200 |
| **Dryopteridaceae** |  |  |  |  |  |  |
| *Cystopteris fragilis* | ALL | Fern | BL, RS1, RS2 |  | Ar, Bo, Br, Ch, Ur | 0-4500 |
| *Woodsia montevidensis* | ALL | Fern | BL, RS1, RS2 |  | Ar, Bo, Ch, Br, Pa, Ur | 0-4000 |
| **Ephedraceae** |  |  |  |  |  |  |
| *Ephedra rupestris* | 1, 3 | Shrub | BL, RS1, RS2 |  | Ar, Bo, Ch | 2000-4200 |
| **Fabaceae** |  |  |  |  |  |  |
| *Adesmia crassicaulis* | 1, 2, 3 | Cushion plant | BL, RS1, RS2 |  | Endemic NWA | 3000-4000 |
| *Adesmia schickendantzii* | 1 | Sub-bush | BL, RS1, RS2 |  | Ar, Bo | 2900-4000 |
| *Astragalus arequipensis* | 1 | Erect herb | RS1, RS2 | Probably confused with other species of the genus in BL. Halloy (1985a) recorded c. 10 spp of Astragalus | Ar, Ch | 3000-4500 |
| *Astragalus bustillosii* | 1, 2, 3 | Prostrate herb | BL, RS1, RS2 |  | Ar, Ch | 3000-4000 |
| *Astragalus cryptobotrys* | ALL | Erect herb | BL, RS1, RS2 |  | Ar, Ch | 3700-4700 |
| *Astragalus flavocreatus* | 1, 2, 3 | Prostrate herb | BL, RS1, RS2 |  | Endemic NWA | 3600-4600 |
| *Astragalus peruvianus* | ALL | Prostrate herb | BL, RS1, RS2 |  | Ar, Bo | 3000-4800 |
| *Astragalus uniflorus* | 1, 2, 3 | Prostrate herb | RS1, RS2 | Probably confused with other species of the genus in BL, especially if they had no flowers during the sampling. Halloy (1985a) recorded c. 10 spp of Astragalus | Endemic NWA | 3000-4500 |
| *Lupinus fiebrigianus* | 2 | Erect herb | BL, RS1, RS2 | No data of elevation for Argentina, yes for Bolivia. | Ar, Bo | 3400- 4200 |
| **Geraniaceae** |  |  |  |  |  |  |
| *Geranium sessiliflorum* | ALL | Rosette | BL, RS1, RS2 |  | Ar, Bo, Ch | 0-5200 |
| **Hypoxidaceae** |  |  |  |  |  |  |
| *Hypoxis decumbens* | 1 | Erect herb | RS1, RS2 | High probability of being new. Found at higher altitude than literature for Argentina (3240m in Bolivia) | Ar, Br, Par, Ur | 0-3240 |
| **Iridaceae** |  |  |  |  |  |  |
| *Mastigostyla mirabilis* | 1 | Erect herb | RS1, RS2 | Possibly not identified in BL if not in flower. | Endemic Tucumán | ̴ 3500 |
| *Olsynium junceum* | 1 | Erect herb | BL, RS1, RS2 |  | Ar | 250-4700 |
| *Sisyrinchium hypsophilum* | 1 | Erect herb | RS1, RS2 | Possibly not identified in BL if not in flower. Found at higher altitude than literature. | Endemic NWA | 3000-3800 |
| **Juncaceae** |  |  |  |  |  |  |
| *Luzula racemosa* | 1, 2 | Erect herb | BL, RS1, RS2 |  | Ar, Bo, Ch | 1500-4700 |
| **Loasaceae** |  |  |  |  |  |  |
| *Caiophora nivalis* | 1, 2, 3 | Rosette | BL, RS1, RS2 |  | Endemic NWA | 3500-4400 |
| *Caiophora rosulata* | 1, 3 | Erect herb | BL, RS1, RS2 |  | Endemic NWA | 300-4800 |
| **Malvaceae** |  |  |  |  |  |  |
| *Acaulimalva nubigena* | 1 | Rosette | BL, RS1 |  | Ar, Bo, Pe | 3000-4500 |
| *Nototriche anthemidifolia* | 2, 3 | Rosette | BL, RS1, RS2 |  | Ar, Bo, Ch | 3500-4800 |
| *Nototriche caesia* | ALL | Rosette | BL, RS1, RS2 |  | Endemic NWA | 3700-4500 |
| *Tarasa tenella* | 1, 2 | Prostrate herb | BL, RS1, RS2 | Found at higher altitude than literature for Argentina (In Bolivia 4200m) | Endemic NWA | 2500-3800 |
| **Montiaceae** |  |  |  |  |  |  |
| *Montiopsis modesta* | ALL | Prostrate herb | BL, RS1, RS2 |  | Ar, Ch | 1900-4000 |
| **Onagraceae** |  |  |  |  |  |  |
| *Oenothera nana* | ALL | Rosette | BL, RS1, RS2 |  | Ar, Bo, Ch | 2500-4500 |
| *Oenothera punae* | 1 | Rosette | BL, RS1 |  | Ar, Bo | 2000-4700 |
| **Oxalidaceae** |  |  |  |  |  |  |
| *Oxalis sp. 1* | 3, 4 | Erect herb | BL, RS1, RS2 |  |  | - |
| **Plantaginaceae** |  |  |  |  |  |  |
| *Plantago sericea ssp. Sericans* | 1, 2 | Rosette | BL, RS1, RS2 |  | Ar, Bo, Ch | 2900-4400 |
| **Poaceae** |  |  |  |  |  |  |
| *Anatherostipa mucronata* | 3 | Non-tussock grass | BL, RS1, RS2 |  | Ar, Ch | 3500-4500 |
| *Deyeuxia cabrerae* | 2 | Tussock grass | RS1 | Probably was re-identified as other Poaceae | Ar, Ch | 3500-4200 |
| *Deyeuxia colorata* | 1 | Tussock grass | BL, RS1, RS2 |  | Ar, Bo | 1700-4400 |
| *Deyeuxia curvula* | 3 | Non-tussock grass | BL | Probably re-identified as other Poaceae (e.g. *D. lagurus*) | Ar, Bo, Ch | 3400-4900 |
| *Deyeuxia deserticola var. deserticola* | 1, 2 | Tussock grass | RS1 | Probably re-identified as other Poaceae | Ar, Bo, Ch | 2800-4900 |
| *Deyeuxia fiebrigii* | 1, 2 | Tussock grass | BL | Probably re-identified as other Poaceae | Endemic NWA | 2600-4300 |
| *Deyeuxia heterophylla* | 1, 2, 3 | Non-tussock grass | BL, RS1, RS2 |  | Ar, Bo, Ch | 3000-4800 |
| *Deyeuxia lagurus* | ALL | Non-tussock grass | BL, RS1, RS2 |  | Endemic NWA | 3800-4700 |
| *Deyeuxia vicunarum* | 1, 2, 3 | Non-tussock grass | BL, RS1, RS2 |  | Ar, Bo, Ch | 3200-4900 |
| *Dielsiochloa floribunda* | 4 | Non-tussock grass | BL, RS1 | Probable re-identified in RS2 as other Poaceae | Ar, Bo, Ch | 4500-5300 |
| *Festuca chrysophylla* | 3 | Tussock grass | BL | Probably re-identified as other Poacea (e.g. *D. cabreae* or *J. leptostachia*). | Ar, Ch | 3500-4500 |
| *Festuca dissitiflora* | 2 | Non-tussock grass | BL, RS1 | Probable re-identified in RS2 as other Poaceae | Ar | 1800-4500 |
| *Festuca nardifolia* | 2, 3, 4 | Non-tussock grass | BL, RS1, RS2 |  | Ar, Ch | 3200-4900 |
| *Festuca orthophylla* | 1, 2 | Tussock grass | BL, RS1, RS2 |  | Ar, Ch | 3000-4800 |
| *Festuca uninodis* | 2, 3 | Tussock grass | BL, RS1, RS2 |  | Endemic NWA | 3600-4500 |
| *Jarava leptostachya* | 1, 2, 3 | Tussock grass | BL, RS1, RS2 |  | Ar, Ch | 1200-4600 |
| *Koeleria kurtzii* | 3, 4 | Tussock grass | BL, RS1, RS2 |  | Ar, Bo, Ch | 500-4500 |
| *Koeleria permollis* | 2 | Non-tussock grass | BL, RS1 | Probable re-identified in RS2 as other Poaceae. Found at higher altitude than literature. | Ar, Bo, Ch, Ur | 0-3900 |
| *Muhlenbergia peruviana* | 1, 2, 4 | Non-tussock grass | BL, RS1 | Probable re-identified in RS2 as other Poaceae. | Endemic NWA | 2600-4500 |
| *Nassella rupestris* | 1, 2, 3 | Non-tussock grass | BL, RS1, RS2 |  | Ar, Ch | 3200-4600 |
| *Poa calchaquiensis* | 1, 2, 3 | Non-tussock grass | BL, RS1, RS2 |  | Ar, Ch | 1000-4200 |
| *Poa lepidula* | 4 | Non-tussock grass | BL, RS2 |  | Ar, Bo, Ch, Pe | 4600-5200 |
| *Poa scaberula* | ALL | Non-tussock grass | BL, RS1, RS2 |  | Ar, Ch | 3200-4300 |
| *Poa humillima* | 2, 3, 4 | Non-tussock grass | BL, RS1, RS2 |  | Ar, Bo, Ch | 4000-4600 |
| *Poa kurtzii* | 1, 3, 4 | Non-tussock grass | BL, RS1, RS2 |  | Ar, Ch | 3200-4700 |
| *Poa lilloi* | 2, 3, 4 | Non-tussock grass | BL, RS1, RS2 |  | Ar, Bo, Ch | 3200-5000 |
| **Portulacaceae** |  |  |  |  |  |  |
| *Calandrinia acaulis* | ALL | Rosette | BL, RS1, RS2 |  | Andean dist., Ar to Ve. | 3300-5200 |
| **Pteridaceae** |  |  |  |  |  |  |
| *Cheilanthes pruinata* | 1, 4 | Erect herb | BL, RS1, RS2 |  | Ar, Bo, Ch | 2000-4800 |
| *Pellaea ternifolia* | 1 | Erect herb | BL, RS1 |  | Ar, Ch | 200-4500 |
| **Rosaceae** |  |  |  |  |  |  |
| *Lachemilla pinnata* | 2, 3 | Prostrate herb | BL, RS1, RS2 |  | Ar, Bo, Ch | 3600-4500 |
| *Tetraglochin cristatum* | 1 | Shrub | BL, RS1, RS2 |  | Ar, Bo, Ch | 3000-4500 |
| *Tetraglochin inerme* | 2 | Cushion plant | BL, RS1, RS2 |  | Endemic NWA | - |
| **Rubiaceae** |  |  |  |  |  |  |
| *Galium plumosum* | 1, 3 | Prostrate herb | BL, RS1 |  | Endemic NWA | 2000-4000 |
| **Solanaceae** |  |  |  |  |  |  |
| *Solanum acaule* | 1, 2, 3 | Prostrate herb | BL, RS1, RS2 |  | Endemic NWA | 2500-4700 |
| **Valerianaceae** |  |  |  |  |  |  |
| *Valeriana pycnantha* | 3, 4 | Rosette | BL, RS1, RS2 |  | Ar, Ch | 3600-4800 |
| **Verbenaceae** |  |  |  |  |  |  |
| *Junellia digitata* | 3, 4 | Cushion plant | BL, RS1, RS2 |  | Ar, Ch | 3200-4500 |
| *Junellia minima* | 1 | Cushion plant | RS1, RS2 | Recorded by Halloy (1985a) | Ar | 2800-4600 |
| **Violaceae** |  |  |  |  |  |  |
| *Viola rodriguezii* | 2, 3, 4 | Rosette | BL, RS1, RS2 |  | Endemic NWA | 3500-4500 |
| *Viola calchaquiensis* | 1 | Rosette | BL, RS1, RS2 |  | Endemic Tucumán | 3000-4000 |
|  |  |  |  |  |  |  |
|  |  |  |  |  |  |  |
|  |  |  |  |  |  |  |
